# DemuxTrans: Transformer and temporal convolution network for accurate barcode demultiplexing in nanopore sequencing

**DOI:** 10.1093/bioinformatics/btaf612

**Published:** 2025-11-25

**Authors:** Liyuan Shu, Deyu Zhuang, Jiao Tang, Junyong Zhao, Wei Shao, Xiaoyu Guan, Daoqiang Zhang

**Affiliations:** Key Laboratory of Brain-Machine Intelligence Technology, Ministry of Education, College of Artificial Intelligence, Nanjing University of Aeronautics and Astronautics, Nanjing, 211106, China; Key Laboratory of Brain-Machine Intelligence Technology, Ministry of Education, College of Artificial Intelligence, Nanjing University of Aeronautics and Astronautics, Nanjing, 211106, China; Key Laboratory of Brain-Machine Intelligence Technology, Ministry of Education, College of Artificial Intelligence, Nanjing University of Aeronautics and Astronautics, Nanjing, 211106, China; Key Laboratory of Brain-Machine Intelligence Technology, Ministry of Education, College of Artificial Intelligence, Nanjing University of Aeronautics and Astronautics, Nanjing, 211106, China; Key Laboratory of Brain-Machine Intelligence Technology, Ministry of Education, College of Artificial Intelligence, Nanjing University of Aeronautics and Astronautics, Nanjing, 211106, China; Key Laboratory of Brain-Machine Intelligence Technology, Ministry of Education, College of Artificial Intelligence, Nanjing University of Aeronautics and Astronautics, Nanjing, 211106, China; Key Laboratory of Brain-Machine Intelligence Technology, Ministry of Education, College of Artificial Intelligence, Nanjing University of Aeronautics and Astronautics, Nanjing, 211106, China

## Abstract

**Motivation:**

Oxford Nanopore Technologies (ONT) direct RNA sequencing (dRNA-seq) offers high-resolution, single-molecule analysis but is hindered by the lack of robust multiplex barcoding methods. Existing approaches struggle to accurately demultiplex raw nanopore signals, failing to capture both local patterns and long-range dependencies. This limitation underscores the requirement for advanced solutions to improve accuracy, efficiency, and adaptability in sequencing workflows. We present DemuxTrans, a hybrid deep learning framework that integrates Multi-Layer Feature Fusion, Transformers, and Temporal Convolutional Networks (TCN) for precise barcode demultiplexing.

**Results:**

DemuxTrans achieves state-of-the-art performance across multiple datasets by effectively balancing local feature extraction, global context modeling, and long-term dependency capture, excelling in metrics such as accuracy, recall and F1-score. These results demonstrate DemuxTrans as a scalable, efficient solution for barcode demultiplexing in nanopore sequencing, enabling precise identification of multiplexed RNA samples and improving throughput in transcriptomic and epigenomic analyses.

**Availability and implementation:**

The code and datasets are publicly available on https://github.com/LiyuanShu116/Demuxtrans

## 1 Introduction

The advent of third-generation sequencing (TGS) technologies has significantly advanced the exploration of complex genomes and transcriptomes, providing unprecedented insights into biological systems (Ardui et al. 2018, Pollard et al. 2018). Unlike earlier sequencing methods, TGS enables the direct sequencing of intact DNA and RNA molecules, eliminating the need for fragmentation or PCR amplification. This break-through minimizes amplification biases and allows for single-nucleotide resolution. Among TGS platforms, Oxford Nanopore Technologies (ONT) has gained particular prominence for its direct RNA sequencing (dRNA-seq) technology. By using protein nanopores and motor proteins to control RNA translocation, ONT facilitates high-resolution, single-molecule analysis (Rang et al. 2018). Its applications span genome assembly (Choi et al. 2020), transcriptome assembly (Fang et al. 2021, Sahlin and Medvedev, 2021), methylation studies (Liu et al. 2021, Tourancheau et al. 2021, Sakamoto et al. 2021), and mutation detection (Goenka et al. 2022, Capraru et al. 2022).

**Figure 1.**
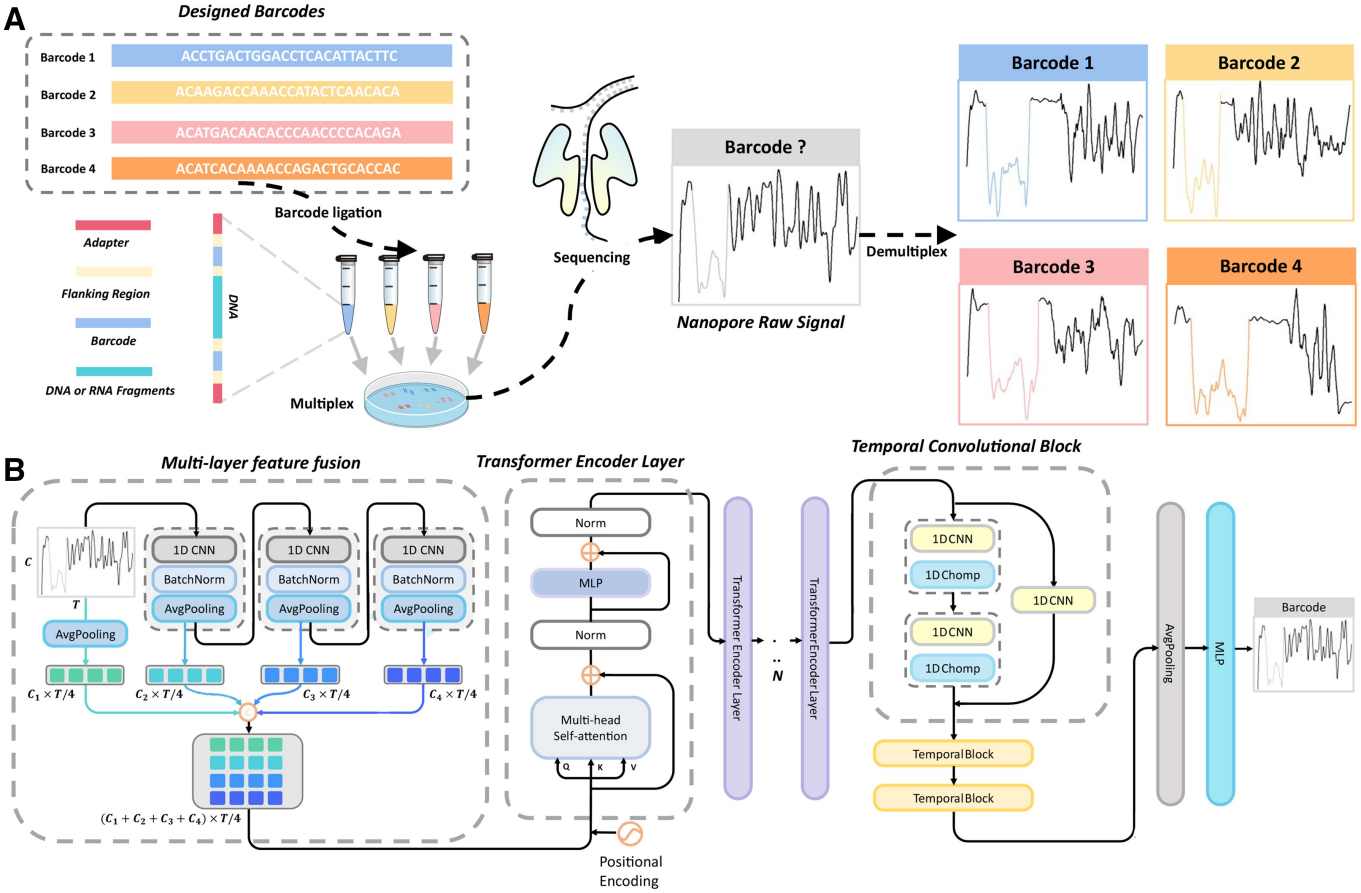
Schematic of multiplex and demultiplex and the proposed DemuxTrans framework. (A) Multiplex and Demultiplex Workflow. DNA or RNA fragments are extracted from each sample, and unique barcode sequences (adapters) are attached to these fragments. These barcodes serve as identifiers for each individual sample. All barcoded samples are pooled into a single mixture for sequencing, generating raw signal data. The barcode signals within each raw signal are detected, and a model is used to demultiplex the barcode signals, assigning them to their respective samples. (B) Architecture of DemuxTrans for Demultiplexing Barcode. A deep learning architecture combining multi-layer feature fusion, Transformer and TCN. The input undergoes 1D CNN-based feature extraction with pooling and normalization, followed by Trans-former layers with multi-head self-attention for long-range dependencies. Temporal convolutional blocks with causal convolutions capture sequential patterns, leading to the final output via pooling and a Multi-Layer Perceptron (MLP).

Despite its transformative potential, the effectiveness of dRNA-seq is limited by the absence of robust molecular barcoding protocols for multiplexing. Multiplex barcoding involves tagging RNA molecules or samples with unique barcode sequences (adapters) before sequencing, allowing multiple samples to be pooled and processed on a single flow cell (Fig. 1A). This greatly improves throughput and reduces cost, especially for low-complexity or low-depth samples (Church and Kieffer-Higgins, 1988). While ONT provides barcoding kits for DNA sequencing (Wick et al. 2017), comparable protocols for direct RNA sequencing remain underdeveloped. The lack of reliable RNA barcoding means that multiplexed dRNA-seq experiments currently suffer from misassignment of reads to samples and decreased overall accuracy, creating a pressing need for improved demultiplexing methods.

Machine learning (ML) techniques have recently become key in genomics for handling complex sequencing data (Le et al. 2020, Auslander et al. 2021), enabling significant improvements in RNA barcode demultiplexing accuracy and efficiency. Current ML-based demultiplexing methods fall into two broad categories: clustering and classification. Clustering-based methods group similar nanopore read signals without barcode labels, exploiting inherent signal patterns to form clusters for distinct samples. For example, Papetti et al. (Papetti et al. 2023) use autoencoders with Self-Organizing Maps to cluster nanopore signals by barcode, and HycDemux (Han et al. 2023) integrates raw signals with basecalled sequences in a GPU-accelerated hybrid clustering algorithm with a voting refinement. Classification-based methods, in contrast, directly assign each raw signal to a barcode class using supervised learning. Typically, a model is trained on labeled examples to map signal features to barcode identities. For instance, QuipuNet (Misiunas et al. 2018) employs 1D convolutional neural networks (CNNs) to extract local signal patterns, Deepbinner (Wick et al. 2018) uses a CNN to scan raw current traces, and DeePlexiCon (Smith et al. 2020) transforms raw signals into images processed by ResNet. More recently, WarpDemuX (Van der Toorn et al. 2024) uses dynamic time warping distance in a support vector machine for fast classification.

However, existing demultiplexing models face notable limitations. Clustering approaches scale poorly and are easily confounded by subtle signal variation and noisy reads, whereas supervised classifiers often struggle to capture long-range dependencies and global temporal contexts. Specifically, CNN-based methods focus on local signal patterns and thus struggle with modeling long-range temporal dependencies. Similarly, DTW–SVM-based methods are sensitive to non-linear time-warping and noise.

In this paper, we propose DemuxTrans, a hybrid framework designed to address these limitations in barcode demultiplexing. Compared to previous approaches, we utilize Transformers (Vaswani et al. 2017) to model global contextual relationships through self-attention mechanisms, and Temporal Convolutional Networks (TCNs) (Lea et al. 2017) to effectively capture long-range temporal dependencies in nanopore time-series signals. DemuxTrans achieves state-of-the-art performance across multiple benchmarks, effectively assigning reads to their correct barcodes with high accuracy, recall, and F1-score, while maintaining competitive efficiency.

In summary, the innovative aspects of DemuxTrans include: (i) a hybrid architecture fusing CNN-based feature extraction with Transformer and TCN modules to capture both local signal details and global temporal structure; (ii) state-of-the-art demultiplexing accuracy on multiple benchmarks, outperforming existing classification and clustering methods; and (iii) improved interpretability through visualization of learned attention and feature patterns, linking model predictions to underlying biological signal characteristics.

## 2 Methods

For the task of demultiplexing barcodes from raw nanopore signals, we propose a deep learning framework specifically designed to process and classify barcode signals, by incorporating several specialized modules. The architecture, as shown in Fig. 1B, comprises three primary components: Multi-Layer Feature Fusion, Transformer Encoder Layer and Temporal Convolution Block (Section 2, available as supplementary data at *Bioinformatics* online). This model is carefully designed to balance local feature extraction, global dependency modeling, and computational efficiency, ensuring robust and reliable performance in handling complex sequential tasks (Section 1, available as supplementary data at *Bioinformatics* online).

### 2.1 Multi-layer feature fusion

The Multi-Layer Feature Fusion module is designed to extract and integrate multi-scale temporal features from time-series data using a stack of convolutional layers, downsampling layers, and residual connections. This module effectively captures hierarchical patterns and fuses information from different feature resolutions to form a rich, multi-scale representation.

#### 2.1.1 Hierarchical convolutional layers

The Hierarchical Convolutional Layers are designed to progressively extract high-level temporal features from the input time-series data while reducing its temporal resolution. This is achieved through a sequence of 1D convolutional layers, each followed by batch normalization, an Exponential Linear Unit (ELU) activation, and average pooling. These operations allow the network to capture both local temporal dependencies and hierarchical feature representations at different resolutions.

The first layer extracts low-level temporal features using a 1D convolution, followed by batch normalization, ELU activation, and average pooling. The output of this layer is given by:


(1) $$x_{1}=\mathrm{AvgPool}(\mathrm{ELU}(\mathrm{BatchNorm}(\mathrm{Conv}1d(X,W_{1}))))$$

where the input *X* is a one-dimensional time-series tensor, $W_{1}$ represents the learnable weights of the first convolutional layer, $x_{1}$ is the output feature map after the first convolutional layer.

Similarly, the second layer extracts intermediate-level features and the final layer extracts high-level features. This process can be expressed as:


(2) $$x_{2}=\mathrm{AvgPool}(\mathrm{ELU}(\mathrm{BatchNorm}(\mathrm{Conv}1d(x_{1},W_{2}))))$$

(3) $$x_{3}=\mathrm{ELU}(\mathrm{BatchNorm}(\mathrm{Conv}1d(x_{2},W_{3})))$$

where $W_{2},W_{3}$ represent the learnable weights of the second and third convolutional layer, $x_{2},x_{3}$ are the output feature map after the second and final layer.

#### 2.1.2 Residual downsampling paths

In addition to the hierarchical convolutional layers, two residual downsampling paths are implemented to preserve low-level features and intermediate representations. A single 1D convolutional layer downsamples the input directly to the intermediate scale:


(4) $$r_{1}=\mathrm{AvgPool}(\mathrm{ELU}(\mathrm{BatchNorm}(\mathrm{Conv}1d(X,W_{r1}))))$$

where $W_{r1}$ is the weight of the downsampling convolutional layer, $r_{1}$ is the downsampled output. The output of the first hierarchical convolutional layer, $x_{1}$, is downsampled using average pooling:


(5) $$r_{2}=\mathrm{AvgPool}(x_{1})$$

These residual paths preserve low-level and mid-level representations that are otherwise degraded through repeated downsampling.

#### 2.1.3 Feature fusion

The outputs from the hierarchical convolutional layers and the residual downsampling paths are concatenated along the channel dimension to form the fused feature representation:


(6) $$F_{\mathrm{fusion}}=\mathrm{Concat}(r_{1},r_{2},x_{2},x_{3})$$

$F_{\mathrm{fusion}}$ aggregates temporal features across multiple levels of abstraction, enabling the model to learn both fine-grained local details and high-level semantic features, which are essential for decoding complex nanopore barcode signals.

## 3 Results

In this section, we compare the performance of DemuxTrans with various state-of-the-art demultiplexing methods. We evaluate DemuxTrans against other classification-based approaches on datasets D1 and D2, compare it to clustering-based approaches on datasets D4–D6, and assess its performance on RNA004 datasets S1–S3. We also present ablation studies to quantify the contribution of each module in DemuxTrans (Section 10, available as supplementary data at *Bioinformatics* online), analyze the sensitivity of DemuxTrans to key hyperparameters, demonstrate its transfer learning capability (Section 11, available as supplementary data at *Bioinformatics* online), and provide visual interpretability of its inner workings (Section 12, available as supplementary data at *Bioinformatics* online).

### 3.1 Datasets and experimental setup

To evaluate the performance of DemuxTrans, we use three groups of benchmark datasets: D1–D3 for comparisons with classification-based methods, D4–D6 for benchmarking against clustering-based approaches, and S1–S3 for assessing performance on RNA004 datasets. Detailed descriptions of these datasets are provided in Section 3, available as supplementary data at *Bioinformatics* online.

Dataset D1 originates from the multiprotein sensing dataset of QuipuNet (Misiunas et al. 2018), which includes individually controlled measurements for specific barcodes. Dataset D2 (Hendra et al. 2022) consists of fast5-format data obtained from ONT sequencing, which are basecalled using ONT’s official Guppy software. Four distinct barcode categories are defined, and the corresponding signals are extracted to create the dataset. We construct dataset D3 using data from the DeePlexiCon training dataset (Smith et al. 2020) to validate the transferability of DemuxTrans. Furthermore, D4 to D6 (Han et al. 2023) incorporate the official nanopore barcodes. Specifically, these datasets are generated using the barcode kits EXP-NBD104, SQK-16S024, and EXP-PBC096, thus enabling evaluation across various barcode complexities. To assess performance on ONT direct RNA sequencing with the SQK-RNA004 chemistry, we construct S1–S3 from raw sequencing runs released with WarpDemuX (Van der Toorn et al. 2024). We ensure that the test sets remain completely unseen during model training and hyperparameter tuning. For fairness in benchmarking, when we compare to other methods, we use the same training and test splits for each method.

The model is implemented using Python 3.11 and PyTorch 2.5, and training is conducted on two Nvidia GeForce RTX 3090 GPUs. Data augmentation techniques are applied to both datasets to enhance the robustness and generalization capabilities of the model. The specific parameter settings are provided in Section 5, available as supplementary data at *Bioinformatics* online.

### 3.2 Comparison results of classification-based methods

The experimental results for classification-based demultiplexing are summarized in Table 1. We evaluate DemuxTrans alongside several baseline classifiers: QuipuNet (Misiunas et al. 2018), Deepbinner (Wick et al. 2018), DeePlexiCon (Smith et al. 2020), and WarpDemuX (Van der Toorn et al. 2024). All models are trained and tested on each dataset separately. The performance is reported in terms of accuracy, precision, recall, F1-score, and test inference time (Section 6.1, available as supplementary data at *Bioinformatics* online).

**Table 1.** Comparison results of different classification methods on dataset D1 and D2.

	D1				D2			
Methods	Accuracy	Recall	F1-Score	Time (min: sec)	Accuracy	Recall	F1-Score	Time (min: sec)
QuipuNet	0.9327	0.9327	0.9330	**00:00.37**	0.9216	0.9216	0.9217	**00:00.93**
Deepbinner	0.9434	0.9434	0.9434	00:00.44	0.9266	0.9265	0.9264	00:01.22
DeePlexiCon	0.8964	0.8964	0.8956	00:03.87	0.7620	0.7620	0.7628	00:22.54
WarpDemuX	0.7523	0.7523	0.7467	05:14.89	0.7356	0.7356	0.7365	05:40.25
DemuxTrans	**0.9529**	**0.9529**	**0.9527**	00:01.03	**0.9431**	**0.9431**	**0.9431**	00:04.88

Best values per dataset are bolded.

On D1, DemuxTrans achieves the highest performance across all evaluation metrics, effectively balancing accuracy, precision, recall, and F1-score. In particular, DemuxTrans attains an accuracy of 95.29% on D1, which is modestly higher than the next best method QuipuNet and substantially higher than the others. This high accuracy is achieved while maintaining favorable computational efficiency: DemuxTrans requires only about 1.03 seconds to process the entire D1 test set, which is only marginally longer than QuipuNet and Deepbinner. Notably, DemuxTrans is significantly faster than the deeper CNN model DeePlexiCon and also faster than WarpDemuX. The underperformance of DeePlexiCon on D1 underscores the limitations of using 2D image transformations for time-series signals, the conversion to images and use of 2D convolution seems to lose important temporal information, leading to lower recall in particular. WarpDemuX, with its kernel-SVM approach, achieves only moderate accuracy on D1. We suspect that the DTW-based kernel, while efficient for small differences, struggles with the subtle signal variability and noise present in D1, resulting in misalignments and misclassifications.

Similarly, for D2, DemuxTrans consistently surpasses other models in accuracy, precision, recall and F1-score. Although DemuxTrans is slightly slower than QuipuNet and Deepbinner, its significantly superior performance more than justifies this modest increase in computational time. DeePlexiCon and WarpDemuX again reveal considerable limitations in computational efficiency, reinforcing the advantage of DemuxTrans in practical scenarios.

**Figure 2.**
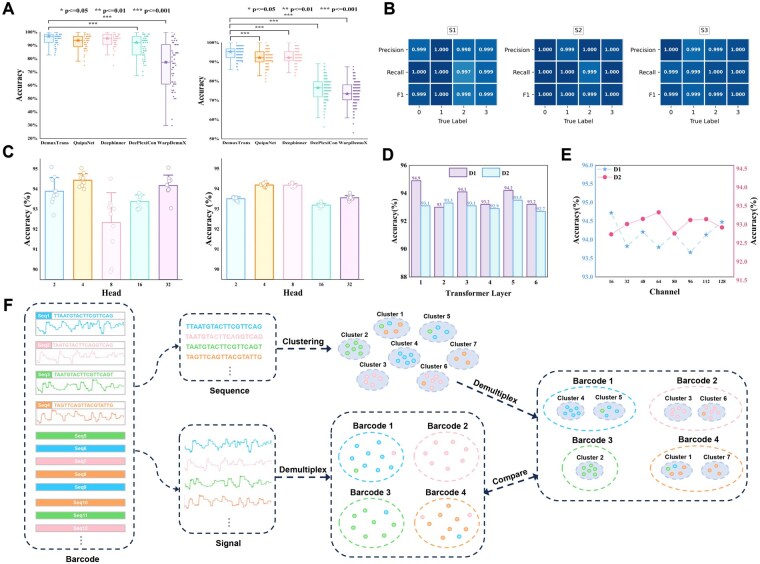
Integrated overview of DemuxTrans evaluation. (A) Accuracy significance analysis of different model performance on dataset D1 and D2. (B) Precision, recall and F1-score metrics for individual barcodes on S1–S3. (C) The influence of the number of multi-head attention heads on the accuracy for different datasets of D1 and D2. (D) The influence of the Transformer layer number on the average accuracy. (E) The influence of TCN dimension on model performance. (F) Schematic of clustering-based versus classification-based barcode demultiplexing workflows on the dataset D4, D5, and D6. Each dataset includes both raw nanopore signal traces and corresponding basecalled barcode sequences. Classification-based methods directly demultiplex barcodes from the signal-level data. In contrast, clustering-based methods group sequences by similarity first, then map each cluster to its dominant barcode label for demultiplexing. The final comparison on the right evaluates the respective outputs from both strategies.

The results presented in Fig. 2A highlight statistically significant differences in the performance of the evaluated models (Section 4, available as supplementary data at *Bioinformatics* online). These results position DemuxTrans as the most effective and reliable model among the five, capable of delivering superior accuracy with high statistical confidence.

### 3.3 Comparison results of clustering-based methods

To evaluate the performance of DemuxTrans against unsupervised clustering methods, we compare it with CD-HIT (Fu et al. 2012), MeShClust v3.0 (Girgis, 2022), MMseqs2 (Kallenborn et al. 2024), and HycDemux (Han et al. 2023). CD-HIT adopts a greedy, identity-based clustering strategy, prioritizing speed and scalability, whereas MeShClust v3.0 integrates a meanshift algorithm to iteratively refine cluster centers based on sequence similarity. MMseqs2 supports two modes: easy-cluster, which uses a cascaded clustering approach to maximize sensitivity, and easy-linclust, which employs a linear-time algorithm optimized for very large datasets. These methods operate on the basecalled sequences and group reads based on sequence similarity. In contrast, HycDemux extracts pseudo-barcode segments from both basecalled reads and raw signals, initializes clusters in nucleotide space, merges and refines them in signal space using GPU-accelerated DTW, and assigns cluster labels by voting against standard barcode signals (Han et al. 2023).

As shown in Fig. 2F, DemuxTrans performs barcode classification directly on raw nanopore signals, whereas CD-HIT, MeShClust v3.0 and MMseqs2 rely on the corresponding basecalled sequences. Consequently, these traditional clustering methods depend substantially on basecalling quality. After clustering, we use a majority voting strategy to map each cluster to its dominant ground truth barcode label (Section 6.3, available as supplementary data at *Bioinformatics* online).

We use homogeneity, completeness, accuracy, and test time to comprehensively assess each method (Section 6.2, available as supplementary data at *Bioinformatics* online). For clustering algorithms, homogeneity and completeness reflect the internal quality of clusters and their effectiveness in fully capturing true categories, while accuracy and runtime enable direct comparisons between DemuxTrans and clustering approaches.

As shown in Table 2 (Section 8, available as supplementary data at *Bioinformatics* online), CD-HIT achieves relatively robust overall performance among the clustering tools when jointly considering homogeneity, completeness, and accuracy, maintaining robust performance over a broad range of identity thresholds. MeShClust v3.0 yields relatively stable results across all datasets, effectively capturing intrinsic sequence structures through iterative refinement, though its meanshift clustering approach results in substantially longer runtimes, particularly on larger datasets. These two operational modes of MMseqs2 exhibit similar accuracy, across the three datasets. Leveraging signal information, HycDemux performs well overall but tends to show lower completeness, consistent with its conservative cluster-merging strategy. Crucially, DemuxTrans achieves the highest demultiplexing accuracy on all three datasets. Despite operating on raw nanopore signal data, thus capturing richer temporal and structural information, its runtime remains competitive.

**Table 2.** Comparison of clustering methods on dataset D4–D6.

Dataset	Method	Identity	Homogeneity	Completeness	Accuracy	Test Time (min: sec)
D4	CD-HIT	0.95	0.801	0.292	0.758	**00:00.29**
	MeShClust v3.0	0.95	0.636	0.261	0.616	00:10.82
	easy-cluster	0.90	0.350	0.172	0.372	00:09.21
	easy-linclust	0.90	0.356	0.176	0.384	00:00.30
	HycDemux	–	0.705	0.233	0.991	00:03.28
	**DemuxTrans**	–	–	–	**0.999**	00:00.98
D5	CD-HIT	0.95	0.996	0.385	0.995	**00:00.50**
	MeShClust v3.0	0.95	0.908	0.380	0.861	00:30.86
	easy-cluster	0.90	0.883	0.379	0.838	00:09.54
	easy-linclust	0.90	0.872	0.376	0.828	00:00.60
	HycDemux	–	0.900	0.299	0.992	00:03.85
	**DemuxTrans**	–	–	–	**0.998**	00:01.44
D6	CD-HIT	0.95	0.999	0.473	0.997	00:04.51
	MeShClust v3.0	0.95	0.928	0.471	0.834	08:06.40
	easy-cluster	0.90	0.995	0.522	0.988	00:10.89
	easy-linclust	0.90	0.995	0.522	0.991	**00:01.94**
	HycDemux	–	0.974	0.460	0.989	00:17.00
	**DemuxTrans**	–	–	–	**0.999**	00:05.04

Best values per dataset are bolded.

### 3.4 Evaluation on RNA004 barcode signal datasets

RNA004 is the latest ONT direct RNA sequencing chemistry, with updated pore and motor components that alter raw signal dynamics compared to earlier kits. Evaluating performance on RNA004 is essential to verify whether DemuxTrans generalizes effectively under changes in current profiles and event statistics. Therefore, we construct three barcode signal datasets S1–S3, using publicly released SQK-RNA004 sequencing data from WarpDemuX. Details of dataset construction are provided in Section 3.5, available as supplementary data at *Bioinformatics* online.

On the S1–S3 datasets, DemuxTrans demonstrates outstanding performance (Fig. 2B). The accuracy, recall, and F1-score for most barcode categories remain within the range of 0.999 to 1.000, with the lowest recall of 0.997 observed only in the S1 dataset. DemuxTrans exhibits no systematic degradation across any barcode set, indicating its ability to robustly distinguish RNA004 barcode signals after training. These results demonstrate that DemuxTrans effectively adapts to the RNA004 chemistry and maintains high accuracy despite the signal distribution shifts introduced by the updated pore and motor.

### 3.5 Hyperparameter parameter sensitivity

The hyperparameters of deep learning models play a critical role in their performance. In this study, we analyze the sensitivity of three key hyperparameters: the number of Transformer heads, the number of Transformer encoder layers, and the number of channels in the TCN.

For dataset D1 and D2 (Fig. 2C), the model performs best with 4 attention heads, while more heads lead to marginal performance declines, likely due to model over-complexity. Fig. 2D illustrates the influence of the number of Transformer layers. A single Transformer layer performs best on D1, while deeper models introduce fluctuations, potentially due to overfitting or training instability in simpler datasets. In contrast, D2 shows less sensitivity to this parameter. The results, illustrated in Fig. 2E, show how varying the TCN channel dimensions impacts accuracy. Since the fluctuation is not substantial, we conclude that changes in the TCN channel dimensions do not significantly impact the model performance.

Based on these observations, we recommend using simpler configurations for low-noise, small-scale datasets and larger capacities for more complex signals. Suggested default settings are summarized in Section 9, available as supplementary data at *Bioinformatics* online.

### 3.6 Visualization of transformer

**Figure 3.**
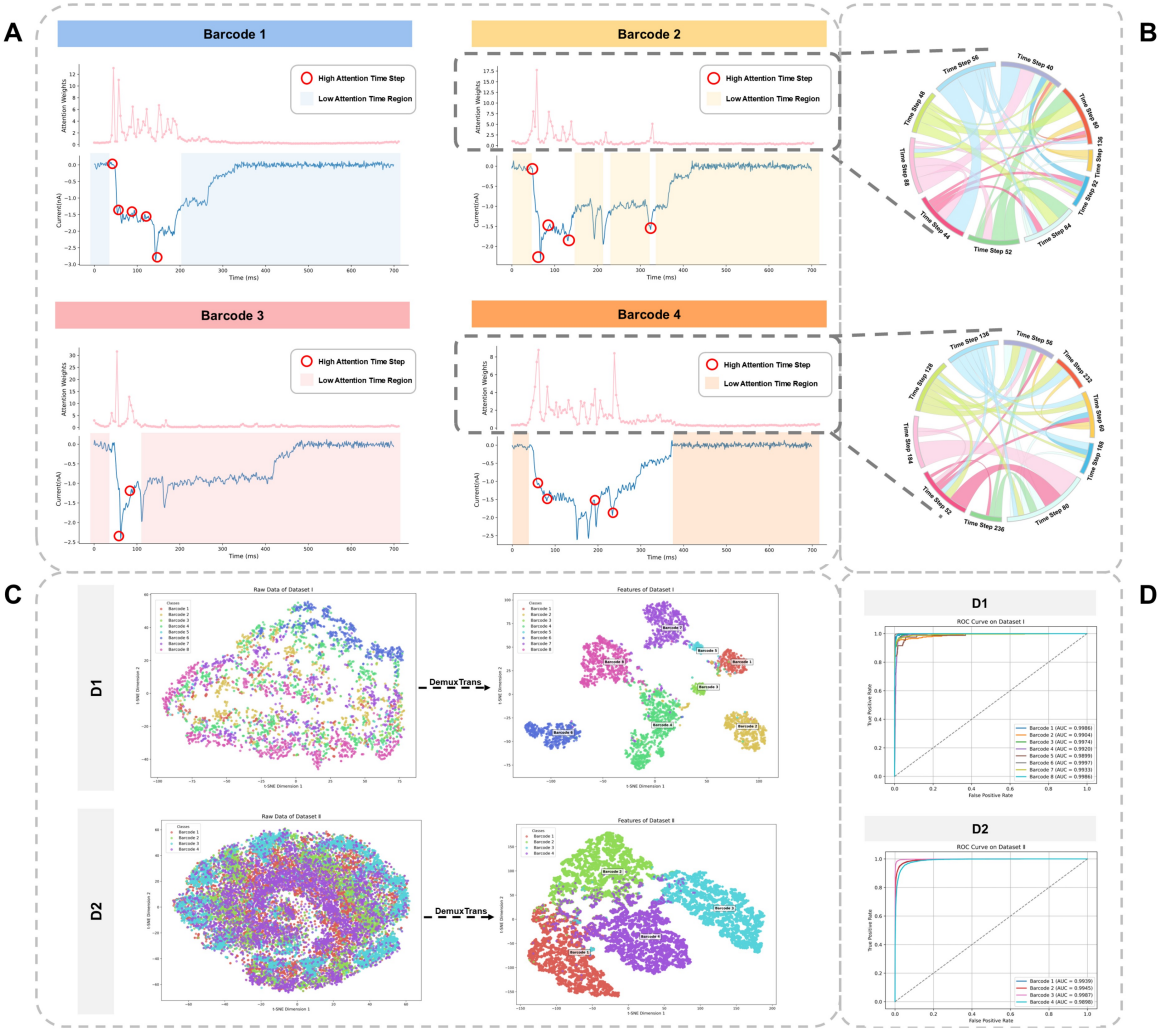
DemuxTrans attention visualization and model performance for barcode demultiplexing. (A) Visualization of DemuxTrans attention maps and raw nanopore sequencing signals for Barcode 1–4. The attention maps highlight the ability to focus on biologically relevant time steps of DemuxTrans, demonstrating how the model detects critical molecular events for accurate barcode classification. (B) Chord diagrams illustrate the inter-timepoint attention relationships for Barcode 2 and Barcode 4. The inter-timepoint dependencies reflect how overlapping nucleotide influences and sequence context effects are integrated by the model, capturing both local and global features of the molecular signal. (C) t-SNE visualizations of feature representations for DemuxTrans in dataset D1 and D2. (D) ROC curves for the model in dataset D1 and D2.

To interpret model behavior, we use Transformer attention maps (Fig. 3A). High-attention regions (red circles) align with significant signal transitions, such as current drops or rises, often corresponding to nucleotide shifts or molecular events (Bao et al. 2021). Low-attention zones align with noise or repetitive regions. Each barcode exhibits distinct attention distributions. For instance, in Barcode 1, the high attention points are concentrated in the initial region of the signal, specifically where a sharp current drop occurs. This suggests that the model relies heavily on molecular events related to the starting sequence of the barcode, such as the entry of the first set of DNA or RNA bases into the sequencing channel. Meanwhile, low attention regions align with noise or less critical parts of the signal, while the attention distribution is dispersed and highly sensitive to peak variations. In Barcode 2, the high attention steps are distributed across multiple regions, particularly between 50 and 150 time points, indicating that this class requires a more comprehensive integration of global signal features for accurate classification. For Barcode 3, attention is primarily focused on a few critical points in the early part of the sequence, where significant signal transitions occur. These high attention points may correspond to key nucleotide shifts or molecular binding events. In contrast, Barcode 4 exhibits a highly distributed attention pattern, reflecting a more complex set of molecular features spread across the sequence.

Nanopore sequencing signals, generated as DNA or RNA molecules pass through the pore, reflect electrical current modulated by each nucleotide and its neighbors (Deamer et al. 2016). This context is essential for decoding sequences and identifying long-range interactions such as RNA structures. Modeling these dependencies reveals complex patterns beyond local features and helps resolve noise and overlapping signals.

The chord diagram in Fig. 3B visualizes inter-timepoint attention weights learned by DemuxTrans, showing how the model connects distinct signal regions. For instance, Barcode 4 exhibits broad attention across the sequence, while Barcode 2 relies on localized dependencies. These patterns illustrate how DemuxTrans integrates both local and global information, enhancing its classification accuracy and offering insights into the biological and technical underpinnings of nanopore signals (Wick et al. 2019).

In t-SNE visualizations (Fig. 3C), raw nanopore signals form overlapping clusters due to noise and variability. After processing with DemuxTrans, barcode-specific features become clearly separable, indicating strong denoising and feature disentanglement. ROC curves (Fig. 3D) further quantify model performance. DemuxTrans achieves AUC values ranging from 0.9899 to 0.9997 on D1 and 0.9898 to 0.9987 on D2, demonstrating consistently high classification accuracy across all barcode classes.

## 4 Discussion and conclusion

Nanopore sequencing enables high-resolution genomic and transcriptomic analysis. However, its multiplexing efficiency is limited by the lack of robust methods for processing raw signal data during barcode demultiplexing. Advances in deep learning provide a promising solution, offering efficient, accurate, and cost-effective demultiplexing.

This study introduces DemuxTrans, a hybrid deep learning framework that combines 1D CNN, Transformers, and TCN to address inefficiencies, lack of robustness, and challenges in capturing long-range dependencies. Our extensive experiments across six datasets demonstrate that DemuxTrans achieves state-of-the-art performance in barcode demultiplexing. It outperforms both cutting-edge supervised classifiers and unsupervised clustering methods on most evaluation metrics. By accurately and efficiently assigning reads to their barcode, DemuxTrans enables the simultaneous sequencing of multiple samples on one flow cell with minimal misassignment. This has practical impact: higher demultiplexing accuracy translates to less contamination between samples, which means one can trust downstream analyses in multi-sample sequencing experiments. Furthermore, efficient demultiplexing ensures that the added complexity of multiplexing does not bottleneck the sequencing workflow.

Another major advantage of our approach is the interpretability provided by attention mechanisms and feature visualization. We demonstrate that attention and Grad-CAM visualizations of DemuxTrans correspond to meaningful features in the nanopore signals, such as current shifts likely caused by specific nucleotide motifs or structural elements. The ability to link signal features to biological processes opens up avenues to extend DemuxTrans or similar models to tasks like detecting modifications or segmenting different regions of a molecule, making our demultiplexing approach broadly useful.

While DemuxTrans achieves strong performance, there remain areas for future improvement. Currently, it operates in an offline mode, classifying reads post-sequencing. However, real-time demultiplexing would enable dynamic sample prioritization during sequencing. Future work will focus on adapting DemuxTrans for on-device streaming, potentially by compressing the model or integrating with ONT APIs to support real-time classification with minimal latency.

In conclusion, DemuxTrans offers an accurate and efficient solution for nanopore barcode demultiplexing. Its hybrid architecture effectively captures both local and global signal patterns, and extensive validation on six datasets confirms superior performance over existing methods. The interpretability enhances transparency and opens the door to further applications, such as barcode design and signal feature discovery. With continued development, DemuxTrans can evolve into a real-time, scalable tool for high-throughput nanopore sequencing. Open-source availability of the model supports broad adoption and future innovation.

## Supplementary Material

btaf612_Supplementary_Data

## Data Availability

All data and implementation details of code can be obtained from github (GitHub - LiyuanShu116/Demuxtrans).
