# Supplementary material for: DemuxTrans: Transformer and temporal convolution network for accurate barcode demultiplexing in nanopore sequencing
Source: Bioinformatics. 2025 Nov 25;41(11):btaf612. doi: 10.1093/bioinformatics/btaf612 (PMC12645835; doi:10.1093/bioinformatics/btaf612)
Supplement: btaf612_Supplementary_Data [file btaf612_supplementary_data.docx]

Supplementary Materials for DemuxTrans: Transformer and Temporal Convolution Network for Accurate Barcode Demultiplexing in Nanopore Sequencing

# 1 Model Architecture

| Table 1. Layer-wise analysis of DemuxTrans | | | |
| --- | --- | --- | --- |
| Layer | Input Shape | Output Shape | Description |
| Input Layer | (batch_size, 1, 700) | (batch_size, 1, 700) | Raw input DNA sequence represented as a single channel with 700 sequence length. |
| Embed | (batch_size, 1, 700) | (batch_size, 175, 256) | Converts input into multi-scale temporal features, integrates information via convolutions. |
| Positional Encoding | (batch_size, 175, 256) | (batch_size, 175, 256) | Adds positional encodings to incorporate token order into the sequence. |
| Transformer Encoder Layer | (batch_size, 175, 256) | (batch_size, 175, 256) | Multi-head self-attention captures global dependencies; feed-forward network enhances features. |
| TCN | (batch_size, 256, 175) | (batch_size, channel, 175) | Captures long-range sequential patterns with dilated convolutions, reducing sequence length. |
| Pooling | (batch_size, channel, 175) | (batch_size, channel, 87) | Applies average pooling to refine features and maintain sequence length. |
| Fully Connected Layers | (batch_size, channel * 87) | (batch_size, classes) | Processes flattened vector through dense layers to produce final classification scores. |

# 2 Methods

## 2.1 Transformer Encoder Layer

The Transformer Encoder Layer is designed to capture global temporal dependencies in the input features using self-attention mechanisms. Since Transformers are inherently permutation-invariant and lack temporal order, a positional encoding $P$ is added to preserve temporal ordering:

$F_{0}=F_{fusion}+P$ (1)

$F_{0}$ is subsequently projected into three matrices: queries $Q$, keys $K$ and values $V$ through linear transformations. The self-attention mechanism then computes the attention weights and the output as follows:

$Attention(Q,K,V)=soft\max(\frac{QK^{T}}{\sqrt{d_{k}}})V$ (2)

Here, $d_{k}$ represents the dimension of $K$.

To further improve efficiency and generalizability, the Transformer Encoder applies multi-head attention (MHA). The outputs from multiple self-attention heads are concatenated and passed through a weight matrix $W^{O}$:

$MHA\left( Q,K,V \right)=Concat({head}_{1},\cdots,{head}_{h})W^{O}$ (3)

After the multi-head attention mechanism, a series of residual connections and layer normalization are performed to facilitate the information flow and stabilize the training process. The output is further processed using the MLP, followed by additional layer normalization, and residual connections, culminating in the final outputs from the multiple Encoder layers.

## 2.2 Temporal Convolution Block

TCN are designed to capture long-range dependencies in sequential data by using causal and dilated convolutions. Unlike traditional convolutions, causal convolutions ensure that the predictions are only based on current and past inputs, maintaining the temporal order of the data.

Dilated convolutions expand the receptive field by introducing gaps between consecutive filter taps. The output for a dilated convolution is:

$y_{t}=\sum_{i=0}^{k-1} w_{i}\cdot x_{t-r\cdot i}$ (4)

where $y_{t}$ is the output at time step $t$, $x_{t-r\cdot i}$ is the input at time step $t-r\cdot i$, $r$ is the dilation rate, $w_{i}$ are filter weights, $k$ is the kernal size.

The TCN model typically consists of multiple layers with varying dilation rates, enabling it to capture both local and long-term dependencies in the data.

# 3 Datasets

We selected six datasets to comprehensively evaluate the performance of our model. Dataset D1 [1] is the complete nanopore signal dataset obtained from QuipuNet, which serves as a comprehensive and diverse benchmark for model evaluation. Dataset D2 [2] consists of concatenated sequencing signals derived from selected reference k-mer sequences that correspond to distinct barcodes. These sequences were carefully chosen to simulate realistic barcode sequencing tasks. Dataset D3 [3], on the other hand, is sourced from DeePlexiCon. To evaluate the performance of our transfer learning approach, we selected a small subset of this dataset for both training and testing. This subset allows us to investigate the effectiveness of our model when applied to limited training data while leveraging transfer learning to enhance generalization. Dataset D4, D5 and D6 [4], obtained from HycDemux, are consist of both raw nanopore signals and their corresponding basecalled sequences.

| Table 2. Datasets description | | | |
| --- | --- | --- | --- |
| Group | Dataset | Origin and Content | Purpose |
| Classification benchmarks | D1 | Complete raw‑signal collection released with QuipuNet. It comprises individually controlled measurements for each barcode and is therefore a diverse, noise‑reduced benchmark for supervised models. | Baseline evaluation of classification methods accuracy and runtime. |
|  | D2 | Concatenated nanopore signals generated from four reference k‑mer barcode sequences. The original FAST5 files were basecalled with Guppy to obtain ground‑truth labels. |  |
|  | D3 | Training data from DeePlexiCon. We randomly extracted a small subset (10 % of the original) for both training and testing. | Probes our transfer‑learning pipeline under limited labelled data. |
| Clustering benchmarks | D4 | HycDemux EXP‑NBD104 set (12 barcodes) containing paired raw signals and base‑called reads. | Baseline evaluation of clustering methods accuracy and runtime. |
|  | D5 | HycDemux SQK‑16S024 set (24 barcodes) with matched signals and sequences. |  |
|  | D6 | HycDemux EXP‑PBC096 set (96 barcodes) with matched signals and sequences. |  |

## 3.1 D1

We adhered strictly to the workflow published by Misiunas, implementing each step with the original QuipuNet utilities and notebooks. First, all eight raw HDF5 archives were concatenated into a single Pandas frame, giving 58178 single-molecule events. We kept only entries flagged Filter=True to remove incomplete or corrupted traces. Each current trace was then resized to a uniform 700 samples. The resampled vector was divided by its channel-specific baseline and sign-flipped, reproducing the amplitude scaling used in the original CNN study. We next computed the RMS noise on every trace beyond sample 20, logging the distribution to parameterize subsequent data‑augmentation routines.

Barcode strings (000-111) were mapped to integers with the native lookup table and converted to 8-way one-hot vectors for supervised training. Following the author’s recommendation, a fixed blind-test subset (3464 events) was carved out by selecting nine specific (barcode, nanopore) pairs; the remaining records were shuffled (seed=42) and split into train (52525) and dev (2189) partitions.

To better understand the characteristics of the datasets, we visualized the raw barcode signals for different classes within each dataset, as shown in Fig. 1.

| 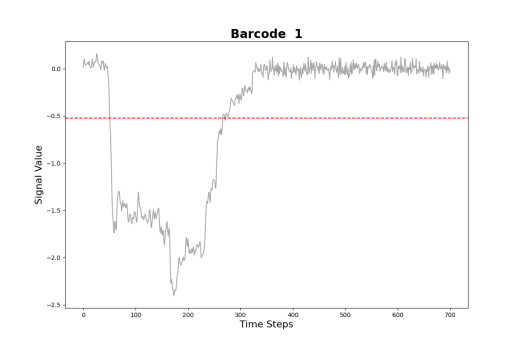 | 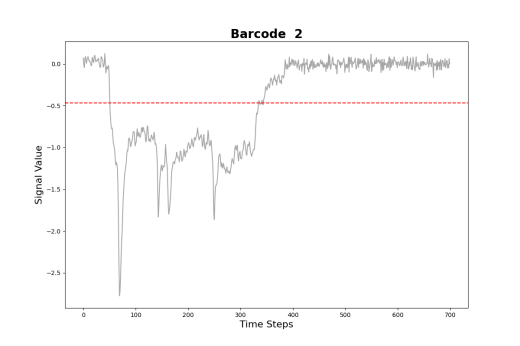 |
| --- | --- |
| 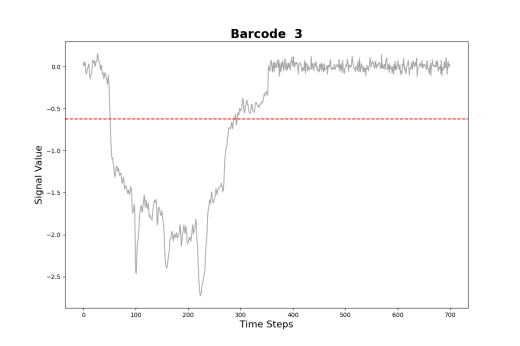 | 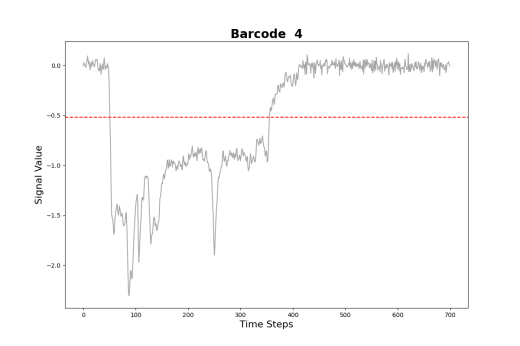 |
| 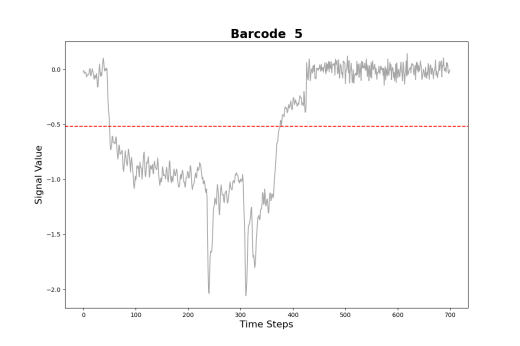 | 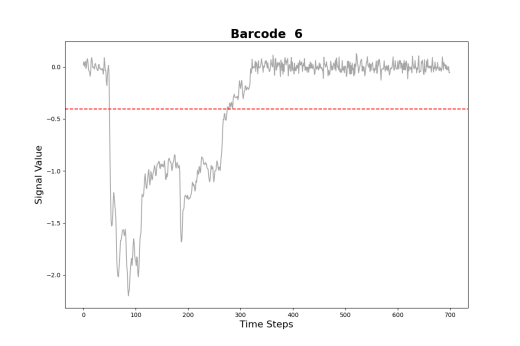 |
| 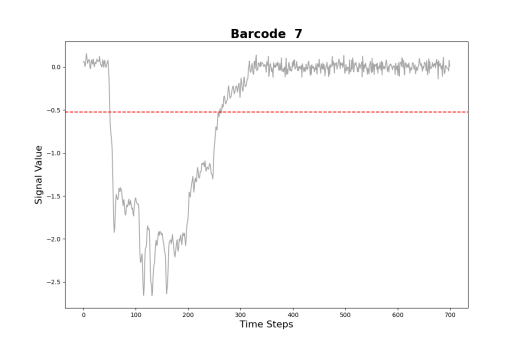 | 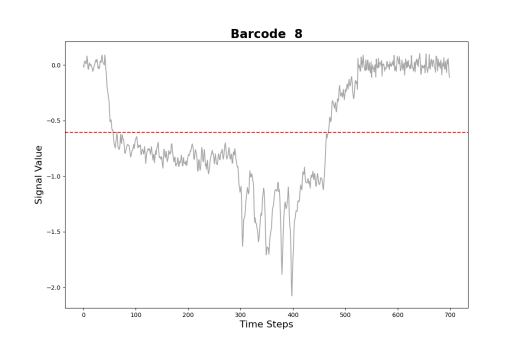 |
| Figure 1. Examples of randomly selected events from the test set of Dataset D1. The red line indicates the signal mean. | |

## 3.2 D2

The raw material for D2 originates from the public FAST5 archive released by Hendra. The original experiment comprises direct RNA runs on an ONT R9.4.1 flow‑cell with a MinION Mk1B sequencer. All FAST5 files were basecalled with Guppy 6.5.7 (sup model) using default high‑accuracy parameters to obtain per-read event tables and reference sequences.

We selected non-overlapping 5-mer motifs, that are well separated in current space and do not occur in adjacent positions within the same read. Each motif defines one barcode class (Barcode 1- Barcode 4) in Table 3, and we visualized the raw barcode signals for different classes within each dataset, as shown in Fig. 2.

| Table 3. Reference 5-mer sequences assigned to each synthetic barcode class in D2 | |
| --- | --- |
| Barcode Class | Constituent 5‑mer sequences |
| Barcode 1 | ACCTG - ACTGG - ACCTC - ACATT - ACTTC - ACAGG - ACTCC - ACCTA - ACTTA - ACTAT - ACTCT - ACCGT |
| Barcode 2 | ACAAG - ACCAA - ACCAT - ACTCA - ACACA - ACTTG - ACCTT - ACAGC - ACAGT - ACTAA - ACCGG - ACTAG |
| Barcode 3 | ACATG - ACAAC - ACCCA - ACCCC - ACAGA - ACTGT - ACTGA - ACTTT - ACAAT - ACCCG - ACACG - ACCGA |
| Barcode 4 | ACATC - ACAAA - ACCAG - ACTGC - ACCAC - ACCCT - ACACC - ACTAC - ACCGC - ACACT - ACATA - ACTCG |

| 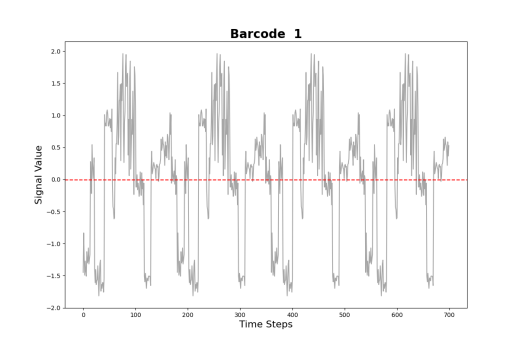 | 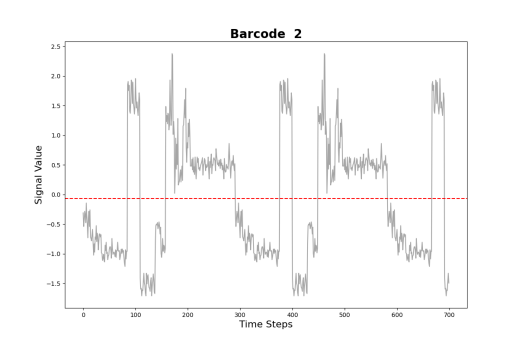 |
| --- | --- |
| 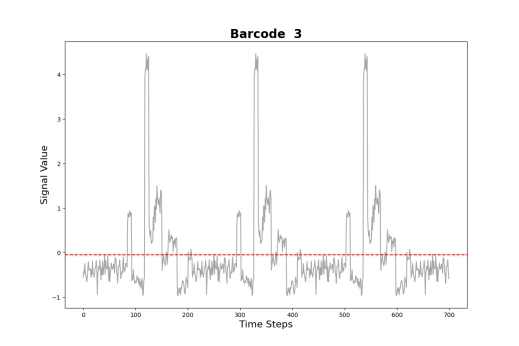 | 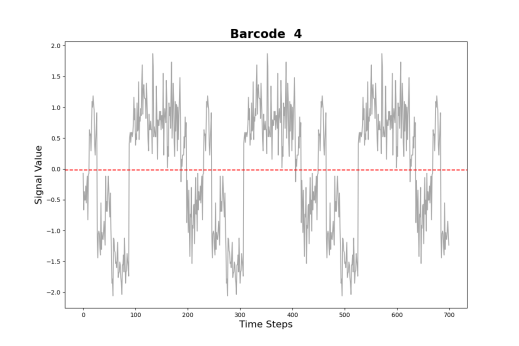 |
| Figure 2.  Examples of randomly selected events from the test set of Dataset D2. | |

## 3.3 D3

The third benchmark set is distilled from the DeePlexiCon training archive released by Smith, which contains native-RNA nanopore reads barcoded with the EXP‑NBD104 kit. We re-processed the raw material at the signal level to obtain a compact, well‑curated corpus suitable for transfer-learning experiments.

The public ZIP bundle was downloaded from the DeePlexiCon GitHub repository. Each entry stores the ionic-current trace (signal), ONT read ID, and the ground-truth barcode label supplied by the authors. All traces were resampled or trimmed to 700 samples using the same routine applied to D1-D2. Traces were divided by the open‑pore baseline estimated over the first 100 samples and sign‑inverted to harmonise polarity with the QuipuNet scale.

D3 is exclusively used to fine-tune models that were first pre-trained on the larger D2 corpus. Its limited size stresses the model’s ability to transfer generic nanopore representations to a new but related barcode distribution when labelled data are scarce.

| 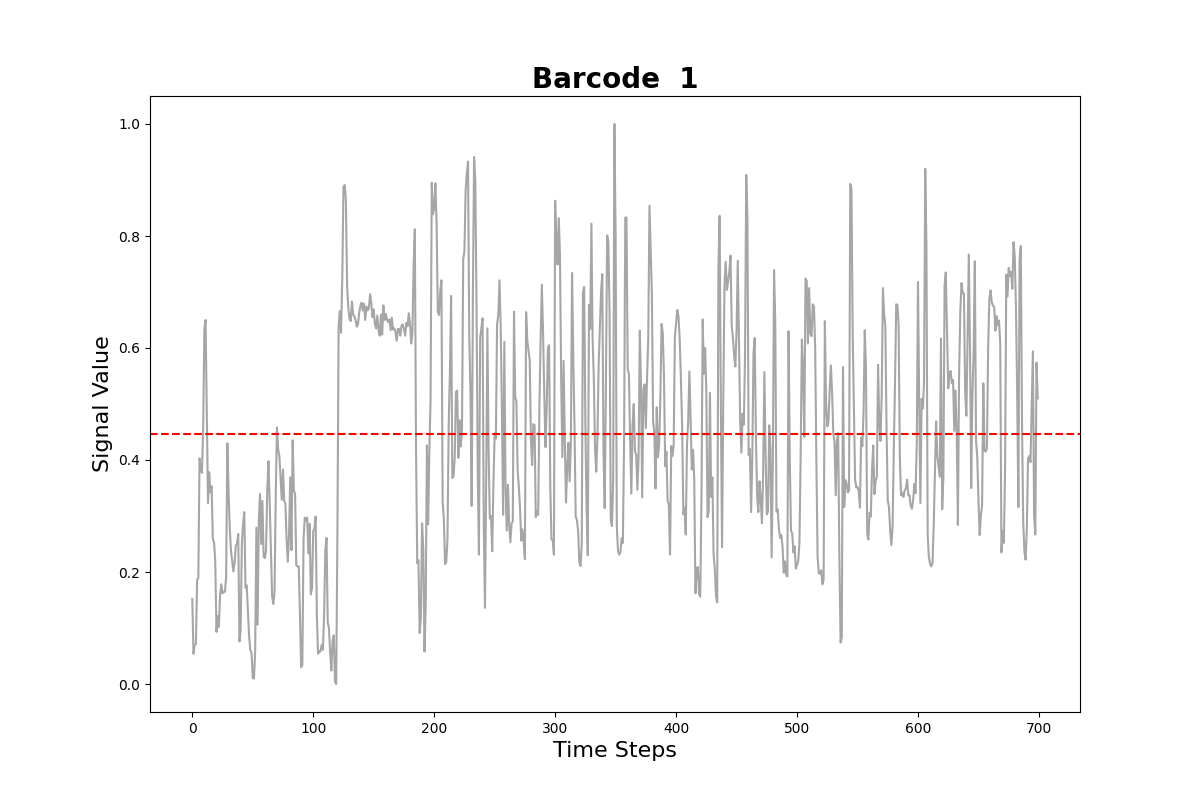 | 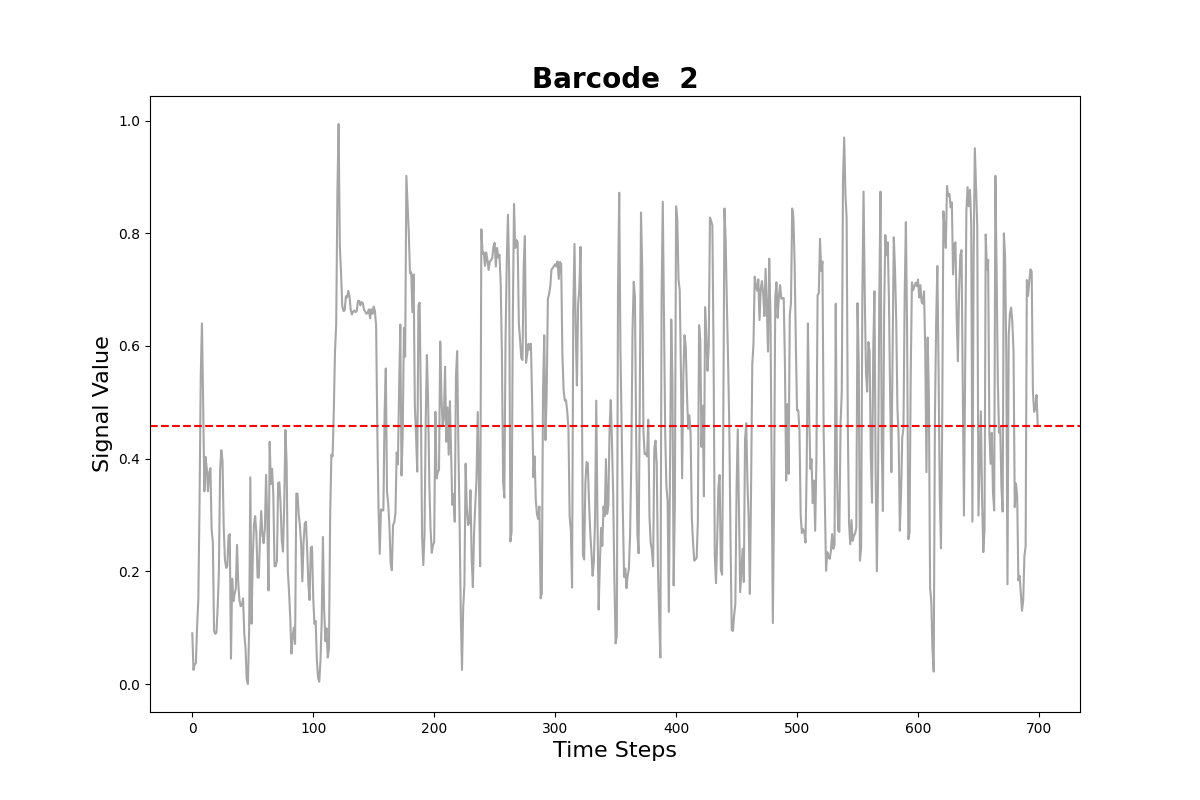 |
| --- | --- |
| 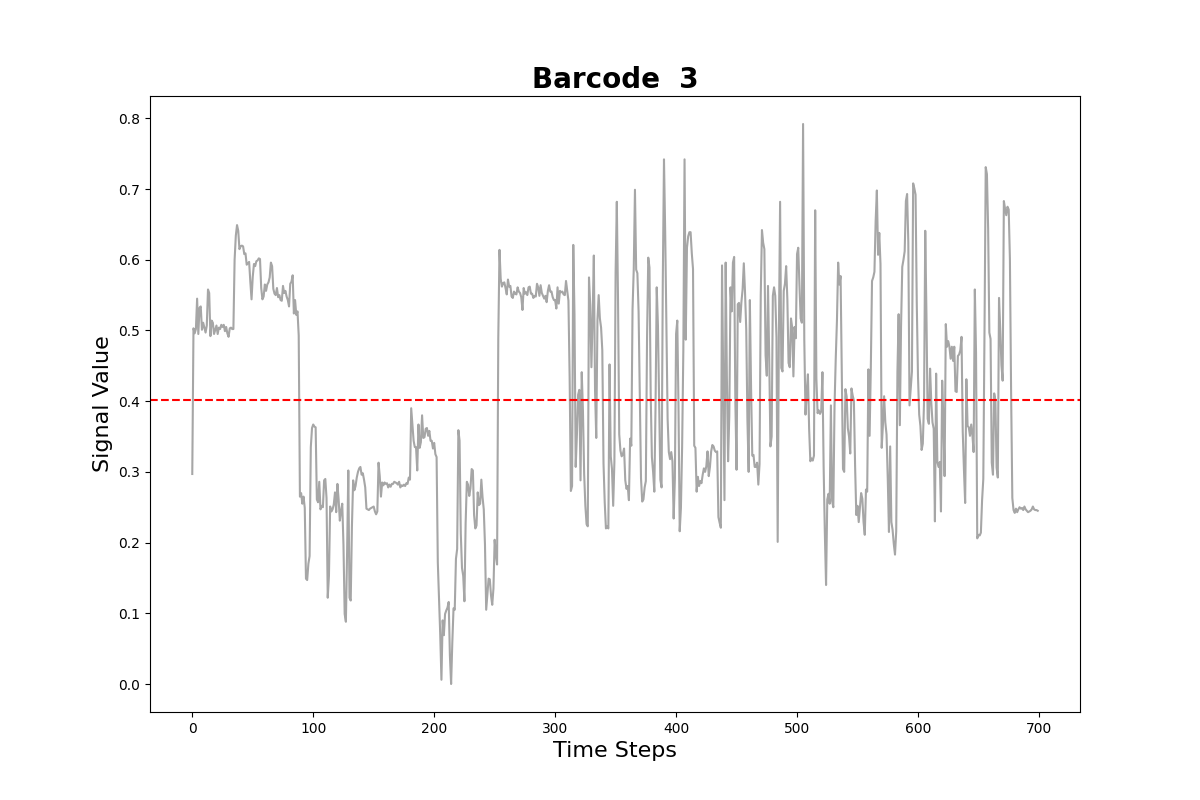 | 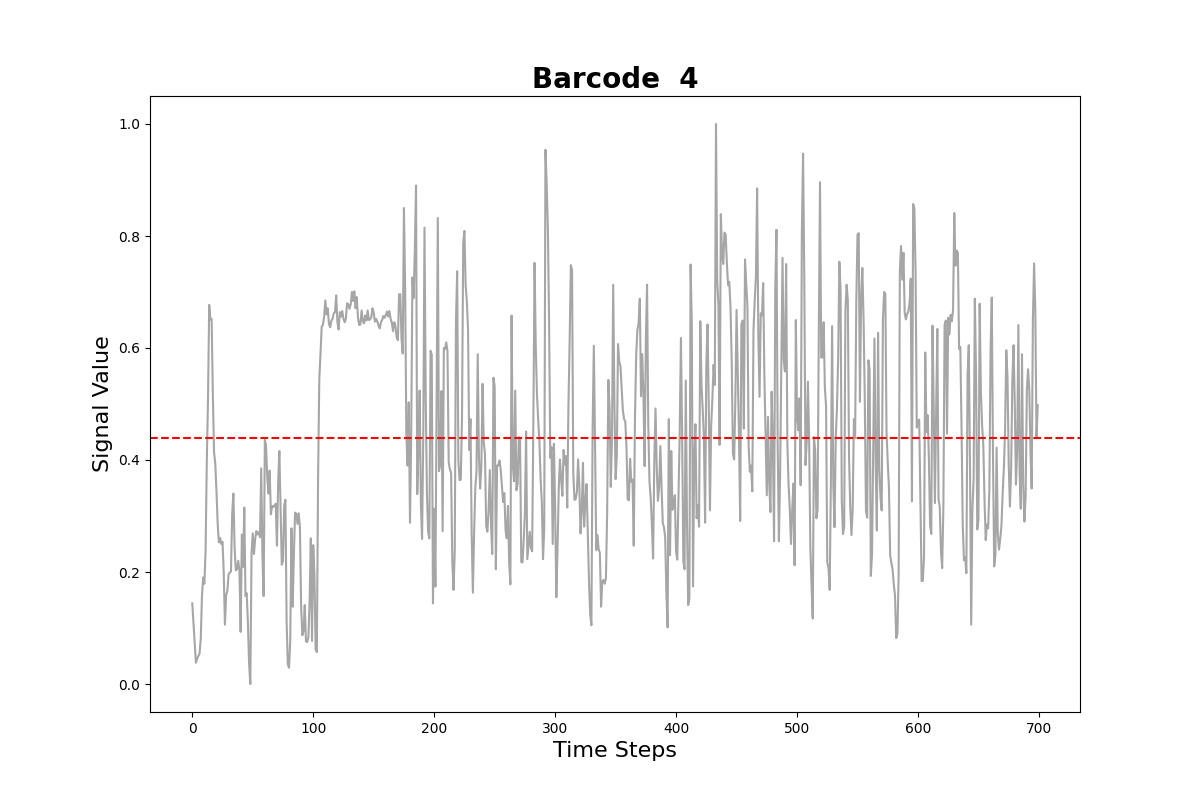 |
| Figure 3.  Examples of randomly selected events from the test set of Dataset D3. | |

## 3.4 D4, D5 and D6

Following the HycDemux workflow, we re-processed the three barcoded nanopore collections (EXP-NBD104, SQK-16S024 and EXP-PBC096) exactly as described by Han and in the project’s public code base, ensuring that every decision point (basecaller model, adapter search window, quality thresholds, split ratio) mirrors the original protocol.

All FAST5 archives and reference FASTA files were downloaded from the HycDemux Zenodo collection. Kit-specific adapter sequences were cross-checked against Oxford Nanopore’s official manuals for EXP-NBD104 (1-12), SQK-16S024 (1-24) and EXP-PBC096 (1-96) to guarantee correct barcode mapping. We generated D4-D6, including both raw nanopore signals and their corresponding basecalled sequences, exactly with the command line pipeline shipped in HycDemux.

## 3.5 S1-S3

We construct three RNA004 barcode signal datasets, S1–S3, from the raw, publicly released SQK-RNA004 direct RNA sequencing runs associated with WarpDemux (ENA accession: PRJEB84366). Following WarpDemuX, we isolate the barcode-bearing adapter signal directly from raw current traces. First, ADAPTed detects the DNA adapter and the DNA/RNA boundary in each read. We then segment the adapter region into discrete events and extract the last 25 events as a fixed-length barcode fingerprint, which is expected to contain the barcode signal.

We generate barcode pseudo-labels by demultiplexing with three released RNA004 models, WDX4_rna004_v0_4_4, WDX4b_rna004_v0_4_6, and WDX4c_rna004_v0_4_6, each targeting a different barcode set. To ensure high-quality labels, we then apply two filters. WarpDemuX includes a dedicated noise category trained specifically for outliers or non-RNA signals (such as channel blockages or boundary detection errors). Sequencing reads assigned to this category will be excluded from evaluation and the dataset. Additionally, we filter reads with confidence scores below 0.9. WarpDemux provides a calibration table containing barcode-specific confidence thresholds, allowing users to set target accuracy levels. For example, on the RNA004 dataset, WarpDemux achieves over 99% accuracy at a confidence threshold of 0.47.

Each of the S1–S3 datasets contains four distinct barcode types. For each type, we randomly sample 10,000 instances for training, and allocate 2,000 instances to both the validation and test sets. These datasets are publicly available at the following Zenodo repository: https://zenodo.org/records/17323295

## 3.6 Dataset Splits

To ensure rigorous evaluation and reproducibility, we adopt a consistent and transparent data partitioning strategy across all datasets. For each benchmark dataset (D1-D6), we divide the samples into non-overlapping training, validation, and test subsets, with proportions tailored to the dataset size and task type.

For supervised classification datasets (D1-D3), we reserve a fixed test set and split the remaining samples into training and validation sets. In D1 and D2, the splits follow an approximate 8:1:1 ratio, while D3, being smaller, is split 1:1 for training and testing.

For clustering benchmarks (D4-D6), where labels are used only for evaluation, we split the available data into training and test sets (8:2), ensuring the clustering methods are tested on the same samples as classification models.

| Table 4. Number of events in training, validation and testing sets in Dataset D1 | | | |
| --- | --- | --- | --- |
| Barcode | Training | Validation | Testing |
| 1 | 5595 | 236 | 253 |
| 2 | 8163 | 340 | 502 |
| 3 | 2334 | 84 | 101 |
| 4 | 15151 | 631 | 827 |
| 5 | 883 | 36 | 83 |
| 6 | 7238 | 313 | 427 |
| 7 | 6458 | 273 | 606 |
| 8 | 6703 | 276 | 665 |
| total | 52525 | 2189 | 3464 |

| Table 5. Number of events in training, validation and testing sets in Dataset D2 | | | |
| --- | --- | --- | --- |
| Barcode | Training | Validation | Testing |
| 1 | 15797 | 5309 | 5257 |
| 2 | 15889 | 5292 | 5338 |
| 3 | 15726 | 5260 | 5216 |
| 4 | 15979 | 5269 | 5319 |
| total | 63391 | 21130 | 21130 |

| Table 6. Number of events in training and testing sets in Dataset D3 | | |
| --- | --- | --- |
| Barcode | Training | Testing |
| 1 | 781 | 795 |
| 2 | 793 | 805 |
| 3 | 801 | 833 |
| 4 | 818 | 761 |
| total | 3193 | 3194 |

| Table 7. Splits of training and testing sets in dataset D4, D5 and D6 | | | |
| --- | --- | --- | --- |
| Dataset | Barcode | Training | Testing |
| D4 | 12 | 2631 | 658 |
| D5 | 24 | 4472 | 1118 |
| D6 | 96 | 20068 | 5018 |

## 3.7 Data Preprocessing

The raw signal data of barcodes serve as the input for our study. Before the data is fed into the neural network, we implement two preliminary steps.

1. Data Normalization:

Given that different barcodes correspond to signals of varying lengths, it is necessary to trim and pad the original signals to obtain signals of uniform length. For signals whose lengths are less than the specified value, we supplement the tail end of the signal with random numbers generated based on the probability density of the Gaussian distribution.

We aim for our model to recognize molecules, not experiments. An issue arises due to the unique shape and conductance of each nanopore, resulting in correlations between measurements taken using the same nanopore. The neural network might overfit to these variations, learning to recognize the nanopore rather than the barcode on the molecule. To mitigate such overfitting, we normalize the length-transformed raw signals of the nanopore using Z-Score normalization. This process retains the dimensional shape of the data while ensuring analytical consistency and can be expressed as:

$x'=\frac{x-\mu}{\sigma}$ (5)

where$x$and$x'$represent the input and output data, $\mu$ and $\sigma$ are the calculated mean and standard deviations.

2. Data Augmentation:

Training signal data often suffers from overfitting, especially when dealing with limited data volumes. To address this challenge in decoding barcode signal data, various data augmentation techniques can be employed to enhance model robustness and generalization. In this study, we adopt three primary augmentation strategies: Baseline Mutation, Duration Mutation, and Noise Addition. Specifically, baseline mutation applies random scaling to the signal values at different temporal positions, with scaling factors drawn from a normal distribution. This introduces variability that simulates baseline shifts in real-world signal acquisition. Duration mutation probabilistically adjusts the duration of temporal points, effectively stretching or compressing portions of the signal to mimic timing variations. Noise addition introduces Gaussian-distributed noise into the signal, simulating random fluctuations and environmental noise. These techniques collectively diversify the training dataset, mitigate overfitting, and improve the model's ability to generalize effectively from a limited number of samples.

# 4 Significance Analysis

We treat the accuracy from each batch for the five models on both D1 and D2 as independent samples. Each batch is considered an independent event, and we perform the Shapiro-Wilk test to assess the normality of these batch accuracies [[5](#ref26)]. Since the data distributions on both datasets do not follow a normal distribution, we adopt the Kruskal-Wallis H nonparametric test [[6](#ref27)], which shows that the two populations differ significantly at the 0.001 level. Furthermore, using Dunn’s test [[7](#ref28)], we observe a significant difference between DemuxTrans and DeePlexiCon, WarpDemuX on dataset D1 ($p\leq0.001$) and a significant difference between DemuxTrans and Deepbinner, QuipuNet, DeePlexiCon and WarpDemuX on dataset D2 ($p\leq0.001$).

| Table 8. Shapiro-Wilk normality test on batch‑wise accuracies | | | | |
| --- | --- | --- | --- | --- |
| Datasets | Methods | W-statistic | p-value | Conclusion ($\alpha=0.05$) |
| D1 | DemuxTrans | 0.89282 | 0.00014 | Reject normality |
|  | QuipuNet | 0.90688 | 0.00044 | Reject normality |
|  | Deepbinner | 0.88693 | <0.0001 | Reject normality |
|  | DeePlexiCon | 0.91706 | 0.00103 | Reject normality |
|  | WarpDemuX | 0.9463 | 0.01721 | Reject normality |
| D2 | DemuxTrans | 0.9607 | <0.0001 | Reject normality |
|  | QuipuNet | 0.96799 | <0.0001 | Reject normality |
|  | Deepbinner | 0.96177 | <0.0001 | Reject normality |
|  | DeePlexiCon | 0.98775 | 0.00678 | Reject normality |
|  | WarpDemuX | 0.9848 | 0.00145 | Reject normality |

| Table 9. Dunn’s post-hoc pairwise comparisons ($\alpha=0.05$, Bonferroni corrected) | | | | | |
| --- | --- | --- | --- | --- | --- |
| Datasets | Comparison | Mean-rank diff | Z-score | p-value | Sig* |
| D1 | DemuxTrans-QuipuNet | 27.58182 | 1.82428 | 0.68109 | 0 |
|  | DemuxTrans-Deepbinner | 15.49091 | 1.02458 | 1 | 0 |
|  | DemuxTrans-DeePlexiCon | 56.04545 | 3.70689 | 0.0021 | 1 |
|  | DemuxTrans-WarpDemuX | 118.51818 | 7.83889 | <0.0001 | 1 |
| D2 | DemuxTrans-QuipuNet | 175.88066 | 4.74611 | <0.0001 | 1 |
|  | DemuxTrans-Deepbinner | 136.22054 | 3.67589 | 0.00237 | 1 |
|  | DemuxTrans-DeePlexiCon | 883.74018 | 23.84759 | <0.0001 | 1 |
|  | DemuxTrans-WarpDemuX | 970.70695 | 26.19438 | <0.0001 | 1 |

Sig* = 1 denotes significant difference at 0.05 level of significance; Sig* = 1 denotes not significant.

# 5 Hyperparameter Settings

For QuipuNet, Deepbinner, and HycDemux, we adopt the authors’ recommended, optimized parameters. For DeePlexiCon, the model’s complexity makes the default settings difficult to train. Therefore, we tune the learning rate to obtain optimal performance. For WarpDemuX (DTW–SVM), which shows substantial parameter sensitivity across datasets, we perform per-dataset tuning of the gamma parameter. For clustering methods (CD-HIT, MeShClust, easy-cluster, and easy-linclust), we optimize the sequence identity thresholds. Details are provided in Supplementary Section 8.

| Table 10. The hyperparameter settings of dataset D1 and D2 | | | |
| --- | --- | --- | --- |
| Category | Hyperparameter | Value | |
|  |  | D1 | D2 |
| Data Processing | Baseline Mutation std | 0.1 | 0.1 |
|  | Duration Mutation std | 0.05 | 0.05 |
|  | Noise Addition std | 0.02 | 0.02 |
| Model Architecture | Embedding Dimension | 256 | 256 |
|  | Number of Heads | 4 | 8 |
|  | Number of Layers | 3 | 3 |
|  | Dropout Rate | 0.1 | 0.1 |
|  | TCN Channels | [512, 256, 128, 48] | [512, 256, 128, 64] |
| Optimization | Learning Rate | 0.0002 | 0.0004 |
|  | Batch size | 64 | 64 |
|  | Weight Decay | 0.02 | 0.02 |
| Training | Epoch | 100 | 150 |

# 6 Evaluation metrics

To evaluate the effectiveness of DemuxTrans and compare it fairly against both classification-based and clustering-based demultiplexing methods, we adopt two metric categories: (1) metrics for supervised classification tasks (such as DemuxTrans, QuipuNet, DeepBinner), and (2) entropy-based metrics for unsupervised clustering tasks (such as CD-HIT, MMseqs2, MeShClust). Each metric is defined below, accompanied by its formula and a detailed explanation of the constituent variables.

To ensure fairness, we respect each tool’s recommended hardware configuration and acceleration. For CPU-only tools, including WarpDemuX, CD-HIT, MeShClust v3.0, and MMseqs2, we execute all operations on the CPU. For GPU-accelerated tools, including HycDemux, QuipuNet, Deepbinner, DeePlexiCon, and DemuxTrans, we utilize GPU acceleration accordingly.

It is important to note that different time measurement strategies are applied depending on the underlying algorithm. For deep learning methods, we measure the time from the processing of the first input batch to the final label output. In contrast, for machine learning tools, we measure the time from the start of the clustering process to the completion of clustering, excluding the time required for mapping cluster assignments to barcode labels.

## 6.1 Classification Metrics

Accuracy measures the proportion of correctly predicted barcode labels across all samples:

$Accuracy=\frac{TP+TN}{TP+FP+FN+TN}$ (6)

TP (True Positives) samples correctly predicted as belonging to a given barcode class, TN (True Negatives) samples correctly predicted as not belonging to that barcode, FP (False Positives) samples incorrectly predicted as that barcode (but are not), FN (False Negatives) samples belonging to the barcode but incorrectly predicted otherwise.

Precision reflects how many predicted barcode assignments are actually correct:

$Precision=\frac{TP}{TP+FP}$ (7)

Recall measures the ability to recover all correct instances of a barcode:

$Recall=\frac{TP}{TP+FN}$ (8)

F1-Score combines precision and recall into a single balanced metric:

$F1-Score=2\cdot\frac{Precision\cdot Recall}{Precision+Recall}$ (9)

Inference time measured in minutes:seconds, this metric records the time required for the model to make predictions on the entire test set using two RTX-3090 GPU. It is crucial for evaluating the efficiency and scalability of demultiplexing methods in high-throughput environments.

TPR (True Positive Rate), also known as recall, represents the proportion of actual positive instances correctly identified by the model. FPR (False Positive Rate) represents the proportion of actual negative instances incorrectly classified as positive. The ROC curve plots the TPR against the FPR at various classification thresholds. The AUC is calculated as the area under the ROC curve. AUC quantifies the overall ability of the model to distinguish between positive and negative classes.

## 6.2 Clustering Metrics

For methods that assign cluster labels without access to ground-truth (e.g., CD-HIT), we use external evaluation metrics based on entropy:

Homogeneity measures whether each cluster contains only members of a single class:

$Homogeneity=1-\frac{H(L|C)}{H(C)}$ (10)

where $L$ is true labels (barcodes), $C$ is predicted clusters, $H(L|C)$ is conditional entropy of true labels given the predicted clusters, $H(L)$ is entropy of the true labels.

Completeness measures whether all members of a class are assigned to the same cluster:

$Completeness=1-\frac{H(C|L)}{H(C)}$ (11)

where $H(C|L)$ is conditional entropy of predicted clusters given the true labels, $H(C)$ is entropy of the cluster assignments.

Identity (for clustering tools like CD-HIT and MMseqs2) is a user-specified threshold that determines how similar two sequences must be to be clustered together. For example, an identity threshold of 0.95 indicates that only sequences with ≥ 95% nucleotide identity will be grouped. It controls the granularity of clusters, and thereby influences the homogeneity and completeness trade-off.

## 6.3 Majority Voting for Clustering Evaluation

This algorithm transforms unsupervised cluster assignments into pseudo-classification outputs by using ground-truth labels derived from sample metadata as anchors. Each cluster is scanned for its member samples, and the most frequent true label among those samples is assigned as the predicted barcode for the entire cluster. This majority-voting scheme ensures that each cluster maps to a single barcode category, allowing the computation of classification metrics such as accuracy.

To clarify the anchor-based majority voting strategy, we present a simplified example demonstrating how cluster-to-label mappings are inferred and applied in practice. Consider a clustering output comprising 8 samples grouped into 3 clusters, each sample having a known ground-truth barcode label. The true labels are derived from sample identifiers using a simple rule (according to HycDemux):

$TrueLabel\left( s \right)=\left\lfloor\frac{s-1}{1000} \right\rfloor$ (12)

this rule implies that sample indices 1001-1999 correspond to barcode 1, 2000-2999 to barcode 2, and so on.

We assign a representative barcode label to each cluster by applying majority voting over the anchor samples it contains. For instance, Cluster A includes three samples from barcode 1 and one sample from barcode 2; therefore, its predicted label is determined as barcode 1. Cluster B consists entirely of two samples from barcode 2 and is accordingly labeled as barcode 2. Cluster C contains two samples from barcode 3 and is thus assigned label 3. Based on this label assignment, only a single sample is misclassified, resulting in a sample-wise classification accuracy of 87.5%.

# 7 Confusion Matrix for Model Evaluation

In this supplementary material, we present the confusion matrices for the models evaluated on two datasets, Dataset D1 and Dataset D2. The confusion matrix provides a detailed view of the model’s performance by comparing the predicted labels with the true labels for each class. It enables the analysis of how well the model is distinguishing between different classes and identifying any systematic misclassifications. For Dataset D1, which contains 8 classes with imbalanced distribution, and Dataset D2, which contains 4 classes with a balanced distribution, we provide the confusion matrices for several models, including DemuxTrans, Deepbinner, DeePlexiCon and QuipuNet. These confusion matrices allow us to analyze each model’s performance across the classes and identify where the model is making errors in classification.

| 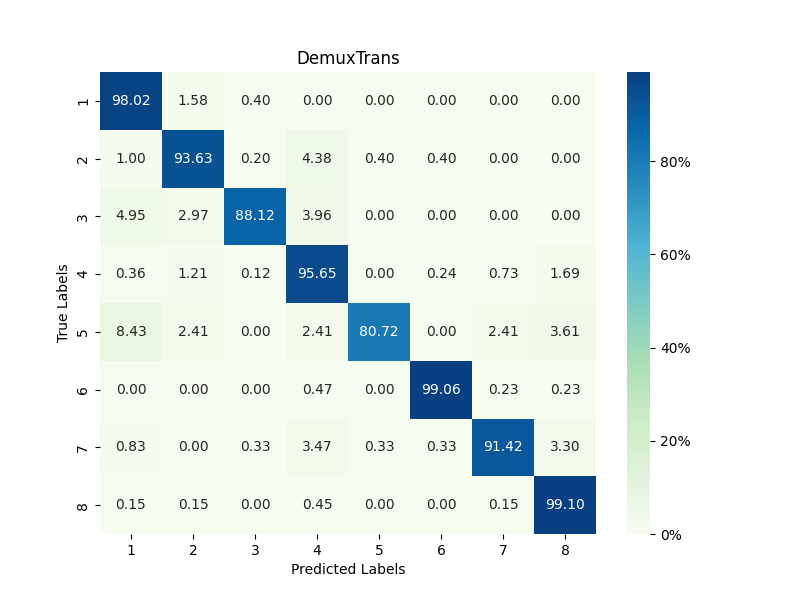 | 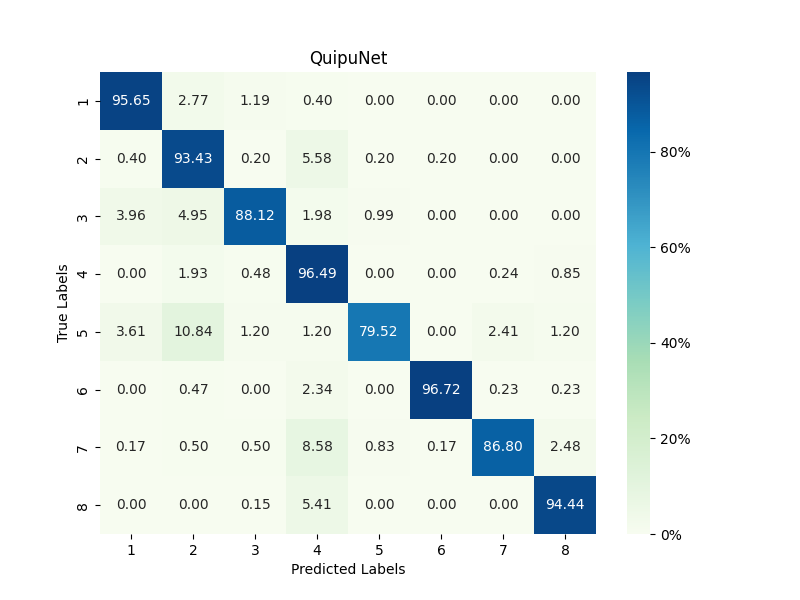 |
| --- | --- |
| 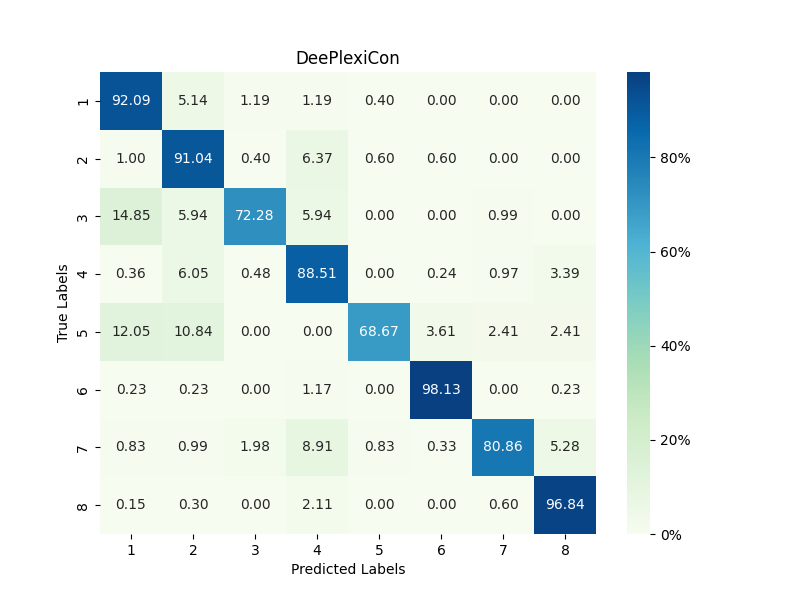 | 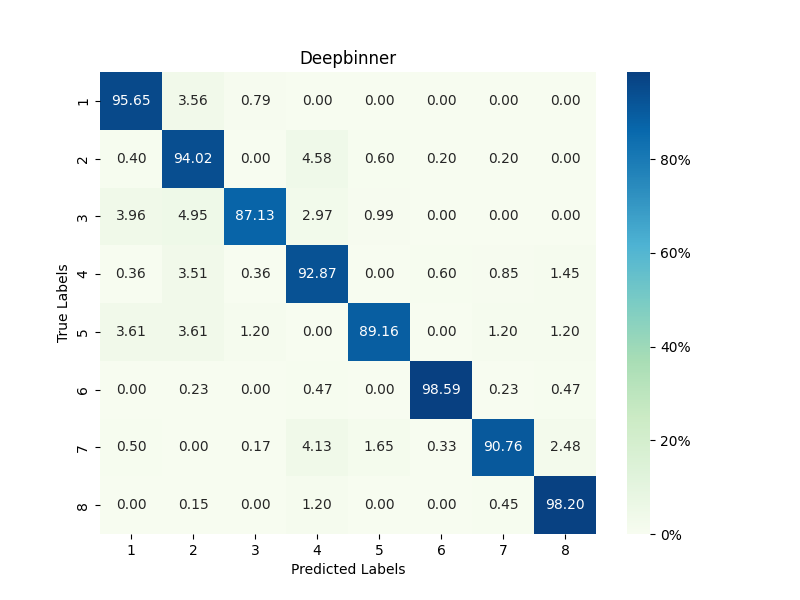 |
| Figure 4. Confusion matrices for DemuxTrans, QuipuNet, DeePlexiCon and Deepbinner models on Dataset D1. | |
|  | |
| 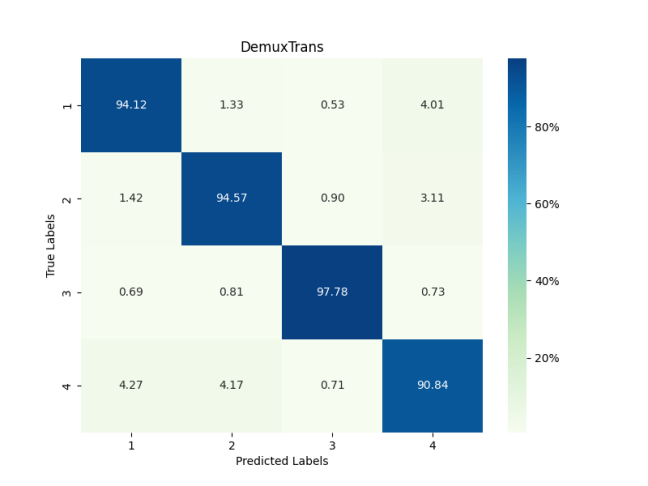 | 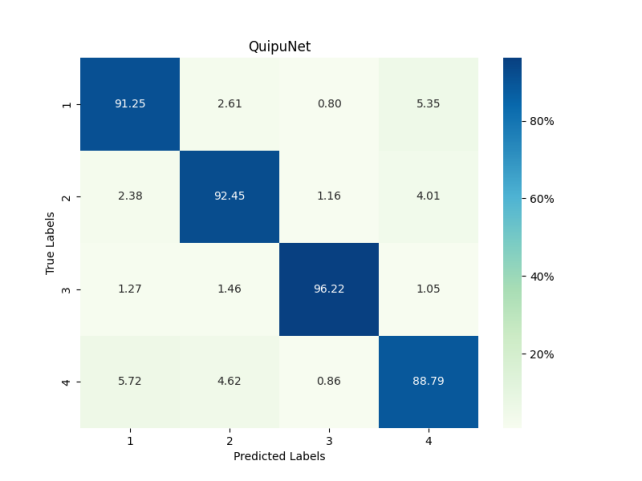 |
| 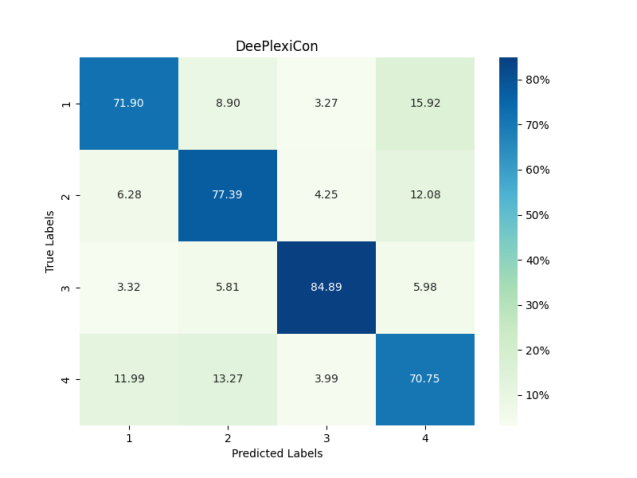 | 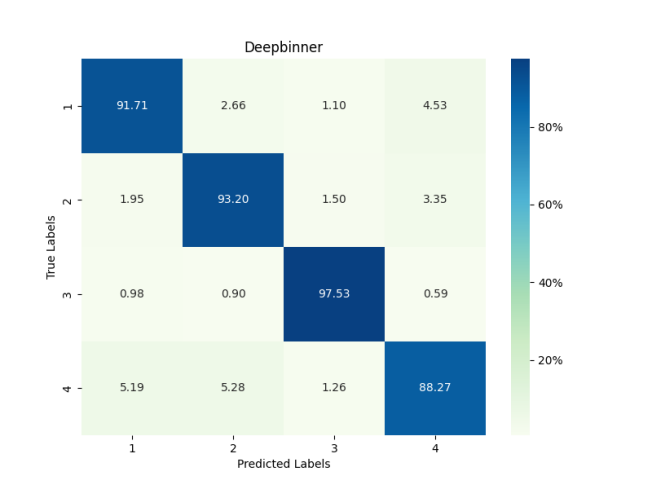 |
| Figure 5. Confusion matrices for DemuxTrans, QuipuNet, DeePlexiCon and Deepbinner models on Dataset D2. | |

# 8 Comparision results of clustering methods

We compare DemuxTrans with CD-HIT [8], MeShClust v3.0 [9], mmseqs_easy_cluster and mmseqs_easy_linclust [10], HycDemux [11]. The command line options for these four clustering tools are listed as follows:

1. CD-HIT: ./cd-hit-est -i infile.fasta -o outfile.fasta -c i
2. MeShClust v3.0: ../bin/meshclust -d input.fasta -o output.txt -t i
3. mmseqs_easy_cluster: mmseqs easy-cluster input.fasta clusterRes tmp --min-seq-id i_1 -c i_2 --cov-mode 1
4. mmseqs_easy_linclust: mmseqs easy-linclust input.fasta clusterRes tmp --min-seq-id i_1 -c i_2 --cov-mode 1

Following the HycDemux workflow, we evaluate the method on the D4–D6 datasets. First, using the reference barcode and adapter sequences, we extract candidate barcode fragments from both raw nanopore current traces and basecalled reads. Next, we apply GPU-accelerated hybrid clustering to these barcode signals and their corresponding sequences to obtain cluster assignments. Finally, we assign barcode labels via the voting procedure using the reference barcode signal templates. For fairness, all evaluations are conducted using HycDemux’s default parameters. An example command-line for D4 is provided below.

1. python mainDataPreparation.py --sigDir D1/D1/ONT12Sigs --seqFile D1/D1/rawSeqsONT12.fasta --sigRootName timeSeries --adapSeq AATGTACTTCGTTCAGTTACGTATTGCT --oADir D1/RealbarcodeONT12AdapterSig --oBDir D1/RealAmpBarSigsONT12 --oTBDir D1/trueBarSigsONT12 --barSeqFile D1/D1/strandONT12BarSeqs.fasta --oBF D1/finalBarSeqsWithLabel.fasta --bl 40
2. python mainHybridClustering.py --barSigDir D1/RealAmpBarSigsONT12/ --barSeqFile D1/finalBarSeqsWithLabel.fasta --sigRootName timeSeries --oclusterFile D1/ONT12_clusteringRes.txt
3. python mainDemultiplexByClusteringRes.py --barSigDir D1/RealAmpBarSigsONT12/ --sigRootName timeSeries --sbarSigDir D1/trueBarSigsONT12/ --clusterFile D1/ONT12_clusteringRes.txt --oDemFile D1/ONT12_DemRes.txt

| Table 11. Comparison results of different classification methods on dataset D4, D5 and D6 | | | | | | |
| --- | --- | --- | --- | --- | --- | --- |
| Datasets | Methods | Identity | Homogeneity | Completeness | Accuracy | Test Time (min: sec) |
| D4 | CD-HIT | 0.80 | 0.270 | 0.217 | 0.340 | 0:00.29 |
|  |  | 0.85 | 0.389 | 0.235 | 0.440 | 0:00.18 |
|  |  | 0.90 | 0.602 | 0.271 | 0.600 | 0:00.22 |
|  |  | 0.95 | 0.801 | 0.292 | 0.758 | 0:00.29 |
|  | MeShClust v3.0 | 0.80 | 0.423 | 0.280 | 0.481 | 0:06.85 |
|  |  | 0.85 | 0.432 | 0.251 | 0.491 | 0:07.69 |
|  |  | 0.90 | 0.521 | 0.246 | 0.554 | 0:07.47 |
|  |  | 0.95 | 0.636 | 0.261 | 0.616 | 0:10.82 |
|  | mmseqs_easy_cluster | 0.60 | 0.184 | 0.110 | 0.238 | 0:09.39 |
|  |  | 0.70 | 0.186 | 0.110 | 0.239 | 0:09.46 |
|  |  | 0.80 | 0.245 | 0.131 | 0.287 | 0:10.37 |
|  |  | 0.90 | 0.350 | 0.172 | 0.372 | 0:09.21 |
|  | mmseqs_easy_linclust | 0.60 | 0.063 | 0.067 | 0.145 | 0:00.26 |
|  |  | 0.70 | 0.111 | 0.084 | 0.186 | 0:00.27 |
|  |  | 0.80 | 0.197 | 0.121 | 0.255 | 0:00.28 |
|  |  | 0.90 | 0.356 | 0.176 | 0.384 | 0:00.30 |
|  | HycDemux | - | 0.705 | 0.233 | 0.991 | 0:03.28 |
|  | DemuxTrans | - | - | - | 0.999 | 0:00.98 |
| D5 | CD-HIT | 0.80 | 0.585 | 0.364 | 0.573 | 0:01.58 |
|  |  | 0.85 | 0.789 | 0.412 | 0.753 | 0:02.45 |
|  |  | 0.90 | 0.957 | 0.420 | 0.939 | 0:02.33 |
|  |  | 0.95 | 0.996 | 0.385 | 0.995 | 0:00.50 |
|  | MeShClust v3.0 | 0.80 | 0.656 | 0.353 | 0.665 | 0:08.65 |
|  |  | 0.85 | 0.685 | 0.352 | 0.670 | 0:10.97 |
|  |  | 0.90 | 0.760 | 0.364 | 0.712 | 0:13.83 |
|  |  | 0.95 | 0.908 | 0.380 | 0.861 | 0:30.86 |
|  | mmseqs_easy_cluster | 0.60 | 0.224 | 0.162 | 0.218 | 0:09.50 |
|  |  | 0.70 | 0.395 | 0.234 | 0.367 | 0:09.29 |
|  |  | 0.80 | 0.700 | 0.337 | 0.655 | 0:09.42 |
|  |  | 0.90 | 0.883 | 0.379 | 0.838 | 0:09.54 |
|  | mmseqs_easy_linclust | 0.60 | 0.220 | 0.175 | 0.223 | 0:00.35 |
|  |  | 0.70 | 0.443 | 0.270 | 0.426 | 0:00.40 |
|  |  | 0.80 | 0.711 | 0.362 | 0.678 | 0:00.50 |
|  |  | 0.90 | 0.872 | 0.376 | 0.828 | 0:00.60 |
|  | HycDemux | - | 0.900 | 0.299 | 0.992 | 0:03.85 |
|  | DemuxTrans | - | - | - | 0.998 | 0:01.44 |
| D6 | CD-HIT | 0.80 | 0.681 | 0.506 | 0.607 | 0:14.70 |
|  |  | 0.85 | 0.932 | 0.599 | 0.895 | 0:36.63 |
|  |  | 0.90 | 0.998 | 0.531 | 0.995 | 0:32.45 |
|  |  | 0.95 | 0.999 | 0.473 | 0.997 | 0:04.51 |
|  | MeShClust v3.0 | 0.80 | 0.528 | 0.417 | 0.446 | 1:57.58 |
|  |  | 0.85 | 0.625 | 0.437 | 0.491 | 2:40.34 |
|  |  | 0.90 | 0.785 | 0.459 | 0.639 | 6:59.06 |
|  |  | 0.95 | 0.928 | 0.471 | 0.834 | 8:06.40 |
|  | mmseqs_easy_cluster | 0.60 | 0.305 | 0.274 | 0.234 | 0:09.83 |
|  |  | 0.70 | 0.578 | 0.390 | 0.481 | 0:10.28 |
|  |  | 0.80 | 0.953 | 0.537 | 0.924 | 0:10.51 |
|  |  | 0.90 | 0.995 | 0.522 | 0.988 | 0:10.89 |
|  | mmseqs_easy_linclust | 0.60 | 0.240 | 0.230 | 0.177 | 0:00.69 |
|  |  | 0.70 | 0.602 | 0.417 | 0.527 | 0:01.03 |
|  |  | 0.80 | 0.953 | 0.572 | 0.928 | 0:01.67 |
|  |  | 0.90 | 0.995 | 0.522 | 0.991 | 0:01.94 |
|  | HycDemux | - | 0.974 | 0.460 | 0.989 | 0:17.00 |
|  | DemuxTrans | - | - | - | 0.999 | 0:05.04 |

# 9 Recommended Hyperparameters

Based on the aforementioned parameter sensitivity experiments, we provide empirical recommendations for hyperparameter selection for end users employing our model, as summarized in Table 10. Simple datasets typically consist of fewer samples, with signals that exhibit minimal noise and a more regular, predictable structure. Conversely, complex datasets usually involve larger sample sizes, higher noise levels, and more irregular or intricate signal structures, which pose greater challenges for feature extraction. These recommendations offer a baseline for model configuration. Users can further fine-tune these settings based on specific dataset characteristics or computational constraints.

| Table 13. Recommended hyperparameter settings for end users | | |
| --- | --- | --- |
| Hyperparameters | Simple Datasets | Complex Datasets |
| Transformer Heads | 4 | 4 |
| Transformer Layers | 1-3 | 2-5 |
| TCN Channels | 16-48 | 48-80 |

# 10 Ablation Experiment

To understand the individual contributions of various components in the DemuxTrans framework, we conduct ablation experiments on dataset D1 and D2, as shown in Table 14. The experiments systematically remove or replace key modules, including Fusion module, the Transformer module, and the TCN module, to evaluate their respective impacts on model performance.

Removing the Fusion module leads to the largest accuracy drop, especially on D2, indicating its importance in extracting multi-scale features essential for complex signal interpretation. The Transformer module, which models long-range dependencies, also causes a notable performance decrease when removed, highlighting its role in capturing contextual information. Similarly, omitting the TCN module results in comparable accuracy loss, confirming that TCN and Transformer offer complementary benefits, local and global temporal modeling, respectively. These results demonstrate that all three components are integral to the overall effectiveness of DemuxTrans.

| Table 14. Ablation experiment on dataset D1 and D2 | | | | |  |  |
| --- | --- | --- | --- | --- | --- | --- |
| Fusion | Transformer | TCN | Accuracy | | |  |
|  |  |  | D1 | D2 | | |
| √ |  |  | 0.8912 | 0.9080 | | |
|  | √ |  | 0.9281 | 0.8749 | | |
|  |  | √ | 0.9402 | 0.8791 | | |
| √ | √ |  | 0.9417 | 0.9293 | | |
| √ |  | √ | 0.9466 | 0.9290 | | |
|  | √ | √ | 0.9483 | 0.9071 | | |
| √ | √ | √ | **0.9529** | **0.9431** | | |

| 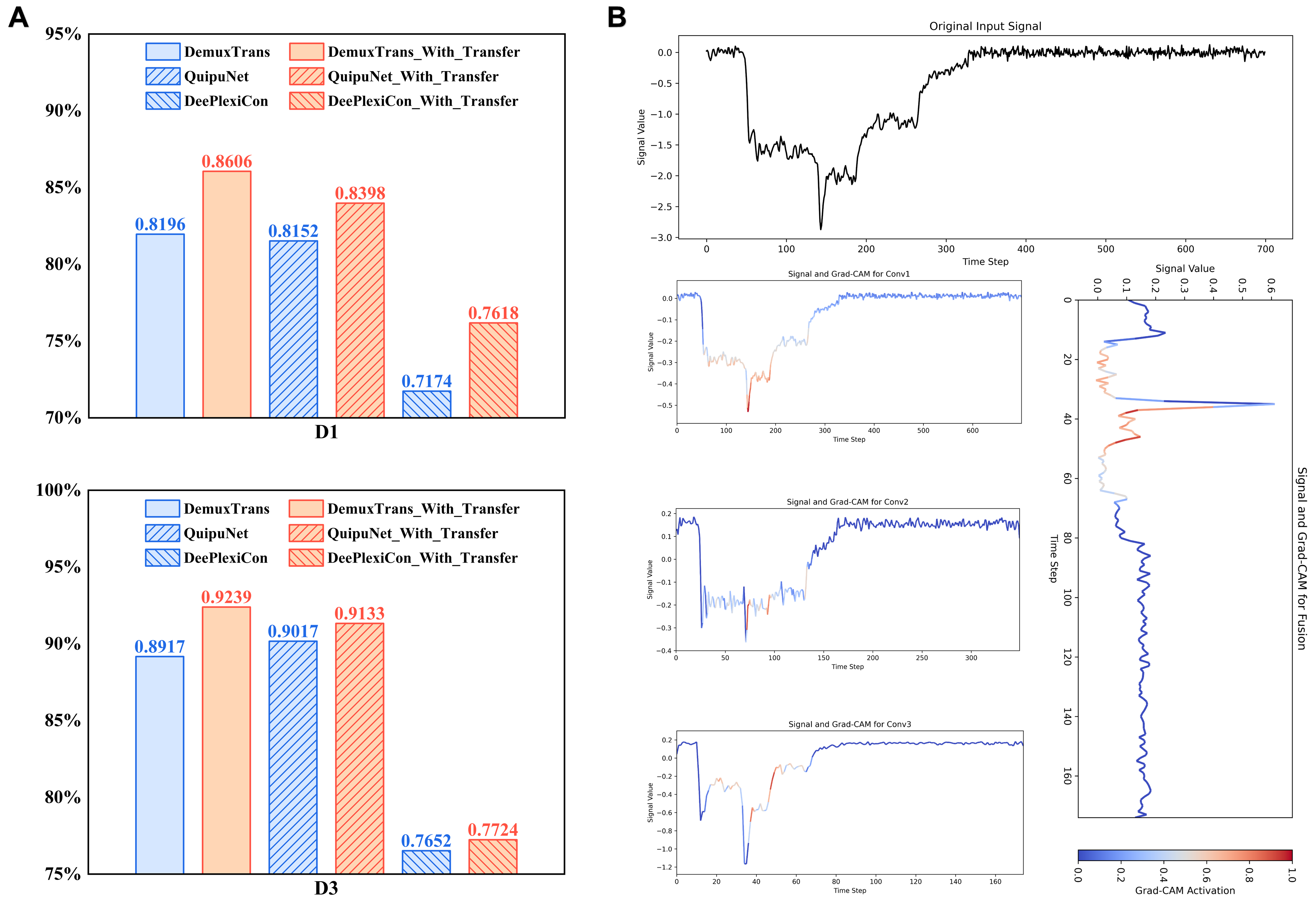 |
| --- |
| Figure 6. (A) Transfer learning performance comparison of DemuxTrans, QuipuNet and DeePlexiCon on dataset D1 and D3. (B) Grad-CAM visualizations of the multi-layer feature fusion module for nanopore barcode demultiplexing. |

# 11 Transfer Learning

Transfer learning is a powerful machine learning technique that leverages knowledge from solving one problem to address a different, yet related, task. Typically, it involves utilizing a model pre-trained on large-scale data to benefit new tasks, substantially reducing the required amount of labeled data and computational resources. This approach is particularly advantageous when labeled data for the target task is limited.

To validate the transferability of the DemuxTrans, we select two representative models for comparison: the QuipuNet model based on 1D CNNs and the DeePlexiCon model based on 2D CNNs. We pre-train all three models on the complete dataset D2. Subsequently, we fine-tune these pre-trained models on dataset D1, using a subset of its training samples, and on dataset D3. The performance results from these transfer learning experiments are illustrated in Fig. 6A.

On dataset D1, before transfer learning, DemuxTrans achieves an accuracy of 81.96\%, slightly higher than QuipuNet and significantly better than DeePlexiCon. After pre-training and subsequent fine-tuning, DemuxTrans demonstrates a notable performance improvement, reaching 86.06\%, thus clearly surpassing the fine-tuned QuipuNet model. DeePlexiCon also benefits markedly from transfer learning, but its overall accuracy remains significantly lower compared to DemuxTrans and QuipuNet, underscoring inherent limitations of the 2D convolutional architecture in capturing temporal nanopore signal features.

On dataset D3, a relatively smaller dataset, the directly trained DemuxTrans exhibits slightly lower accuracy than QuipuNet, likely due to DemuxTrans greater model complexity requiring larger data volumes to generalize effectively. However, following transfer learning, DemuxTrans achieves superior accuracy, significantly outperforming the fine-tuned QuipuNet. This improvement highlights the effectiveness of DemuxTrans in leveraging pre-trained knowledge from larger datasets. DeePlexiCon, despite benefiting from transfer learning, continues to demonstrate limited overall performance due to its structural limitations.

These results demonstrate that DemuxTrans exhibits superior transferability, significantly benefiting from pre-training on large datasets. This capability makes DemuxTrans particularly robust and advantageous for barcode demultiplexing tasks where labeled data availability is constrained.

# 12 Visualization of Multi-Layer Feature Fusion

To further elucidate the function of the multi-layer feature fusion module, we use the Grad-CAM method to visualize the distribution of attention weights along the temporal current signal. In Fig. 6B, the top black curve represents the raw current signal obtained directly from nanopore sequencing. This signal typically exhibits low-frequency drift, random noise, and abrupt current transitions caused by changes in k-mer combinations.

In the lower left panels, the Grad-CAM visualization of the first convolutional module shows distinct red activation regions that coincide with clear current transitions in the barcode signal. These transitions occur as short k-mer sequences pass rapidly through the nanopore, indicating that this layer primarily captures local, high-frequency features. The second convolutional module focuses on broader temporal patterns and shows reduced sensitivity to transient current jumps, suggesting that it integrates local variations into mid-scale k-mer combination patterns. The third convolutional module yields a smoother Grad-CAM map and accentuates critical transition points, which demonstrates that this layer suppresses noise while emphasizing features that are crucial for discrimination.

The vertical panel on the right presents the output after the fusion of features from these convolutional modules. This fused representation not only retains local transient information but also amplifies activation at key time steps along the entire signal. Such a fusion strategy enables the model to capture both the fine-grained details and the overall temporal structure of the barcode signal, thereby improving the demultiplexing performance.

# References

1. Misiunas K, Ermann N, Keyser UF. QuipuNet: convolutional neural network for single‑molecule nanopore sensing. Nano Lett 2018;18:4040–5.
2. Hendra C, Pratanwanich PN, Wan YK, et al. Detection of m6A from direct RNA sequencing using a multiple‑instance learning framework. Nat Methods 2022;19:1590–8. https://doi.org/10.1038/s41592‑022‑01666‑1
3. Smith MA, et al. Barcoding and demultiplexing Oxford Nanopore native RNA sequencing reads with deep residual learning. Genome Res 2020;30:1345–53. https://doi.org/10.1101/gr.260422.119
4. Han R, Qi J, Xue Y, et al. HycDemux: a hybrid unsupervised approach for accurate barcoded sample demultiplexing in nanopore sequencing. Genome Biol 2023;24:222. https://doi.org/10.1186/s13059‑023‑03048‑0
5. Shapiro SS, Wilk MB. An analysis of variance test for normality (complete samples). *Biometrika* 1965;**52**:591–611. https://doi.org/10.2307/2333709
6. Kruskal WH, Wallis WA. Use of ranks in one‑criterion variance analysis. *J Am Stat Assoc* 1952;**47**:583–621. https://doi.org/10.1080/01621459.1952.10483441
7. Dunn OJ. Multiple comparisons using rank sums. *Technometrics* 1964;**6**:241–52. https://doi.org/10.1080/00401706.1964.10490181
8. Fu L, Niu B, Zhu Z, Wu S, Li W. CD‑HIT: accelerated for clustering the next‑generation sequencing data. *Bioinformatics* 2012;**28**:3150–2. https://doi.org/10.1093/bioinformatics/bts565
9. Girgis HZ. MeShClust v3.0: high‑quality clustering of DNA sequences using the mean‑shift algorithm and alignment‑free identity scores. *BMC Genomics* 2022;**23**:423. https://doi.org/10.1186/s12864‑022‑08646‑2
10. Kallenborn F, Chacon A, Hundt C, et al. GPU‑accelerated homology search with MMseqs2. *bioRxiv* 2024;2024.11.13.623350. https://doi.org/10.1101/2024.11.13.623350
